# Brain Inositol Is a Novel Stimulator for Promoting *Cryptococcus* Penetration of the Blood-Brain Barrier

**DOI:** 10.1371/journal.ppat.1003247

**Published:** 2013-04-04

**Authors:** Tong-Bao Liu, Jong-Chul Kim, Yina Wang, Dena L. Toffaletti, Eliseo Eugenin, John R. Perfect, Kee Jun Kim, Chaoyang Xue

**Affiliations:** 1 Public Health Research Institute Center, University of Medicine and Dentistry of New Jersey, Newark, New Jersey, United States of America; 2 Department of Microbiology, Molecular Genetics and Immunology, University of Kansas Medical Center, Kansas City, Kansas, United States of America; 3 Tianjin Research Center of Basic Medical Science, Tianjin Medical University, Tianjin, China; 4 Division of Infectious Disease, Department of Medicine, Duke University Medical Center, Durham, North Carolina, United States of America; 5 Department of Microbiology and Molecular Genetics, University of Medicine and Dentistry of New Jersey, Newark, New Jersey, United States of America; University of Birmingham, United Kingdom

## Abstract

*Cryptococcus neoformans* is the most common cause of fungal meningitis, with high mortality and morbidity. The reason for the frequent occurrence of *Cryptococcus* infection in the central nervous system (CNS) is poorly understood. The facts that human and animal brains contain abundant inositol and that *Cryptococcus* has a sophisticated system for the acquisition of inositol from the environment suggests that host inositol utilization may contribute to the development of cryptococcal meningitis. In this study, we found that inositol plays an important role in *Cryptococcus* traversal across the blood-brain barrier (BBB) both in an *in vitro* human BBB model and in *in vivo* animal models. The capacity of inositol to stimulate BBB crossing was dependent upon fungal inositol transporters, indicated by a 70% reduction in transmigration efficiency in mutant strains lacking two major inositol transporters, Itr1a and Itr3c. Upregulation of genes involved in the inositol catabolic pathway was evident in a microarray analysis following inositol treatment. In addition, inositol increased the production of hyaluronic acid in *Cryptococcus* cells, which is a ligand known to binding host CD44 receptor for their invasion. These studies suggest an inositol-dependent *Cryptococcus* traversal of the BBB, and support our hypothesis that utilization of host-derived inositol by *Cryptococcus* contributes to CNS infection.

## Author Summary

*Cryptococcus neoformans* is an AIDS-associated human fungal pathogen that annually causes over 1 million cases of meningitis world-wide, and more than 600,000 attributable deaths. *Cryptococcus* often causes lung and brain infection and is the leading cause of fungal meningitis in immunosuppressed patients. Why *Cryptococcus* frequently infects the central nervous system to cause fatal meningitis is an unanswered critical question. Our previous studies revealed a sophisticated inositol acquisition system in *Cryptococcus* that plays a central role in utilizing environmental inositol to complete its sexual cycle. Here we further demonstrate that inositol acquisition is also important for fungal infection in the brain, where abundant inositol is available. We found that inositol promotes the traversal of *Cryptococcus* across the blood-brain barrier (BBB), and such stimulation is fungal inositol transporter dependent. We also identified the effects of host inositol on fungal cellular functions that contribute to the stimulation of fungal penetration of the BBB. We propose that inositol utilization is a novel virulence factor for CNS cryptococcosis. Our work lays an important foundation for understanding how fungi respond to available host inositol and indicates the impact of host inositol acquisition on the development of cryptococcal meningitis.

## Introduction

*Cryptococcus neoformans* is a basidiomycetous yeast pathogen that often causes life-threatening infections. It causes the most common fungal infection of the central nervous system (CNS) in HIV-infected persons and may present as encephalitis, meningitis, or cerebral-space-occupying lesions [1], [2], [3], [4], [5], [6]. Cryptococcal CNS infections are uniformly fatal in the absence of treatment [1], [7]. A recent survey suggests that each year there are around 1 million new cases of cryptococcal meningitis, which result in over 600,000 deaths annually [2]. Despite its medical importance and significant research efforts [3], [8], [9], [10], the molecular basis of cryptococcal CNS infection and host factors affecting disease development are poorly understood, which complicates efforts for rapid diagnosis and effective treatment. Hence, there is an urgent need to understand the molecular basis of cryptococcal CNS infection to allow the discovery and development of safer and more effective antifungal drugs.

*C. neoformans* is a globally ubiquitous organism, which is commonly associated with certain environmental niches, including plants and soil contaminated with plant debris and bird droppings. Our previous studies revealed that this fungus can utilize inositol from plant surfaces to complete its sexual cycle [11]. Inositol is essential for cellular structure and regulation of intracellular signaling in all eukaryotes. Recent studies showed that the enzymes involved in inositol metabolism and inositol sphingolipid biosynthesis play a central role in the pathogenesis of *C. neoformans* [12], [13]. The inositol phosphorylceramide synthase 1 (Ipc1) protein, an enzyme of the fungal sphingolipid pathway, activates protein kinase C (PKC), which regulates the cell wall integrity of *Cryptococcus* and manifestation of its virulence factors [12], [14], [15]. Moreover, although it prefers to grow on media containing fermentable sugars such as glucose, *C. neoformans* can utilize free inositol as a sole carbon source [16], [17]. Consistent with the central importance of inositol in its development and virulence, *Cryptococcus* has developed a sophisticated inositol acquisition system that contains an unusually large inositol transporter gene (*ITR*) family with more than ten members, which contrasts with the one or two members found in most other fungi [18], [19], [20], [21]. We also demonstrated that these *ITR*s are required for cryptococcal infection in murine models [11], [22], [23]. In addition, *Cryptococcus* can utilize intracellular glucose to produce inositol in a multi-step de novo inositol biosynthetic pathway in which inositol 1-phosphate synthase (Ino1) is the rate-determining enzyme [19], [24].

*Cryptococcus* invasion and traversal of the blood-brain barrier (BBB) are prerequisites for CNS infection, the major cause of morbidity and mortality in people with cryptococcosis. There are evidences for both direct invasion of the endothelial cells lining the brain vasculature [25], [26] and for a “Trojan horse” mechanism whereby cryptococci enter the CNS after macrophage ingestion [27], [28], [29]. Several factors, including urease [30], [31], phospholipase B1 [32], [33], as well as host plasmin [34], have been reported to be involved in the *Cryptococcus* invasion of the BBB. It was recently reported that *Cryptococcus* interacts with lipid rafts of human brain microvascular endothelial cells (HBMECs) to promote invasion in a glycoprotein CD44-dependent manner [35]. Hyaluronic acid produced by the fungus has been found to function as a ligand for the CD44 receptor during the fungal-host cell interaction [36], [37]. However, the molecular basis for the highly frequent occurrence of cryptococcal CNS infection remains poorly understood. Human and animal brains contain high concentrations of free inositol, and inositol can be used as a carbon source for *Cryptococcus*. Inositol is one of the most abundant metabolites in the human brain; it is located mainly in glial cells, and functions as an osmolyte. Inositol is present in the human cerebellum (5.1 mM) at 200-fold higher concentrations than in plasma (0.02 mM) [38]. Even higher inositol concentrations (>8 mM) are detected in astrocytes that directly associate with the BBB, and inositol can be rapidly released during hyperosmolarity [38], [39]. It is believed, because of the tight interaction of astrocytes with brain microvascular endothelial cells of the BBB, that the inositol concentration around the BBB is much higher than in plasma, although the inositol level in the parenchyma around cerebral vasculature has not been precisely determined. Together with the importance of *ITR*s in fungal infection, we hypothesize that brain inositol is an important host factor for the development of cryptococcal meningitis. We further hypothesize that fungal inositol transporters are important for sensing and/or transporting host inositol during disease progression.

In this study, we utilize an *in vitro* human BBB model and *in vivo* murine models to dissect the role of fungal inositol transporters and host inositol in the traversal of *Cryptococcus* across the BBB and in the development of cryptococcal meningitis. Our results showed that addition of inositol can facilitate *Cryptococcus* transmigration in an *in vitro* BBB model in an *ITR*-dependent manner. These observations tie inositol to the fungal infection in the brain. This work provides a framework explaining the role of inositol in enabling *Cryptococcus* to cross the BBB.

## Results

### Inositol enhances *C. neoformans* transmigration across an HBMEC monolayer

**Figure 1:**
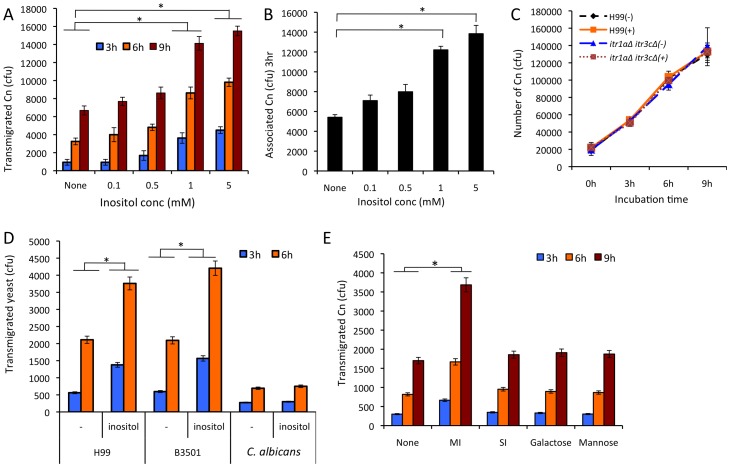
*Myo*-inositol stimulates the traversal of *Cryptococcus* across the HBMEC monolayer. (A and B) Cryptococcal transmigration and association assays were done with an *in vitro* human BBB model generated with HBMEC as described in Materials and Methods. The indicated concentrations of myo-inositol were added to the bottom compartment of Transwells before addition of 10^5^ *C. neoformans* (Cn) cells to the top compartment. The number of *Cryptococcus* cells transmigrated was determined by plating the medium collected from the bottom compartment after 3, 6, and 9 hr incubation (A), while the association was determined after 3 hr incubation (B). (C) Wild type H99 and its *itr1aΔ itr3cΔ* double mutant were resuspended in HBMEC culture medium and grown at 37°C in the absence or presence of 1 mM inositol. After 3, 6, and 9 hr incubation, the number of viable *Cryptococcus* cells was determined by measuring colony-forming units (CFU) on blood-agar plates. (D) Transmigration assays were done with *C. neoformans* strains H99 and B3501, and *Candida albicans* strain (ATCC90028) in the absence or presence of 1 mM inositol in the bottom compartment. (E) Transmigration assays were performed in the presence of different monosaccharides. The equal concentration (1 mM) of myo-inositol (MI), scyllo-inositol (SI), galactose or mannose was added to the bottom compartment prior to addition of *C. neoformans*. These experiments were independently performed three times. Error bars indicate the standard deviations. “*” indicates statistical significance (P<0.001).

Previous studies demonstrate that the route by which *C. neoformans* gains access to the CNS is through traversal across the BBB [37], [40], [41]. The high abundance of inositol in human brain is suggested to be a host factor that promotes the high rate of cryptococcal meningitis [22], [42]. To understand whether inositol plays a role in the development of cryptococcal CNS infection, we investigated the role of free inositol in *C. neoformans* transmigration across the BBB, using an *in vitro* human BBB system. Our *in vitro* BBB system is composed of human brain microvascular endothelial cells (HBMECs) grown on Transwell membranes to confluence, separating the top compartment (blood side) and bottom compartment (brain side) as described in Materials and Methods. We initially performed transmigration assays with *C. neoformans* in the presence of inositol. Inositol was added to the bottom compartment of the Transwells 30 min prior to addition of *Cryptococcus* cells to the top compartment to mimic the situation in the brain. As shown in Fig. 1A, the number of *C. neoformans* var. *grubii* (strain H99) cells that transmigrated across the HBMEC monolayers was not different from control transmigration (no inositol) at inositol concentrations up to 0.5 mM. However, at inositol concentrations greater than 1 mM, transmigration of *C. neoformans* was increased 3-fold compared to controls without inositol treatment, indicating that inositol enhances cryptococcal traversal in a dose-dependent manner.

Because the binding of fungal cells to HBMEC monolayers is the first step in transmigration, association assays were carried out under the same inositol treatment. The results showed a significantly better association between cryptococcal cells and the brain endothelial cells when the bottom chamber contained 1 mM or higher concentration of inositol, indicating that inositol can promote *Cryptococcus* binding (Fig. 1B).

Because the tissue culture medium for HBMEC is rich in nutrients including sufficient glucose (8 mM) for optimal fungal growth, all *Cryptococcus* strains should grow well in this medium. To address the concern that fungal cells may proliferate better in the presence of additional inositol, which might account for the apparent increase in fungal cells in the bottom compartment in the presence of high inositol, the growth rate of H99 in the bottom culture medium was determined in the presence or absence of 1 mM inositol. The results showed that H99 proliferated at the same rate with or without inositol (Fig. 1C). The average replication time for H99 in both media was about 2.2 hours. Therefore, the increased number of *Cryptococcus* cells in the bottom compartment at higher inositol levels could not be due to increased proliferation, and must be the result of increased transmigration. Thus, these results demonstrate that inositol stimulates traversal of cryptococcal cells across the BBB.

### The effect of inositol on fungal transmigration is *Cryptococcus* specific

To determine whether the inositol effect is strain specific, the transmigration assay was also carried out with *C. neoformans* var. *neoformans* strain B3501. *C. neoformans* var. *grubii* strains are in general more virulent than var. *neoformans* strains even though both varieties are able to cause systemic cryptococcosis and meningitis [43]. The number of transmigrated B3501 cells was comparable to that of strain H99, demonstrating that inositol enhances transmigration of *C. neoformans* regardless of strain origins (Fig. 1D). In addition, the inositol effect on transmigration was examined in *Candida albicans*, another yeast pathogen that occasionally crosses the BBB through direct transcytosis to cause CNS infection in humans [44], [45]. *C. albicans* contains one inositol transporter, Itr1, that is not required for fungal virulence [20]. Transmigration of *C. albicans* occurred at a much lower rate and was not enhanced by inositol (Fig. 1D).

Subsequently, the transmigration assays were performed in the presence of another inositol isomer scyllo-inositol, or other monosaccharides such as galactose and mannose. Myo-inositol elevated the efficiency of cryptococcal transmigration exhibiting 2-fold greater number of transmigrated fungal cells than the untreated control after 3, 6 and 9 hrs of incubations (Fig. 1E). However, the cryptococcal transmigration remained unchanged in the presence of other sugars. This result indicates that myo-inositol is a specific effector for promoting cryptococcal traversal across the BBB. Taken together, our findings indicate that inositol specifically increases BBB traversal by *C. neoformans*.

### *Cryptococcus*-HBMEC interactions lead to increased inositol permeability of the HBMEC monolayer

**Figure 2:**
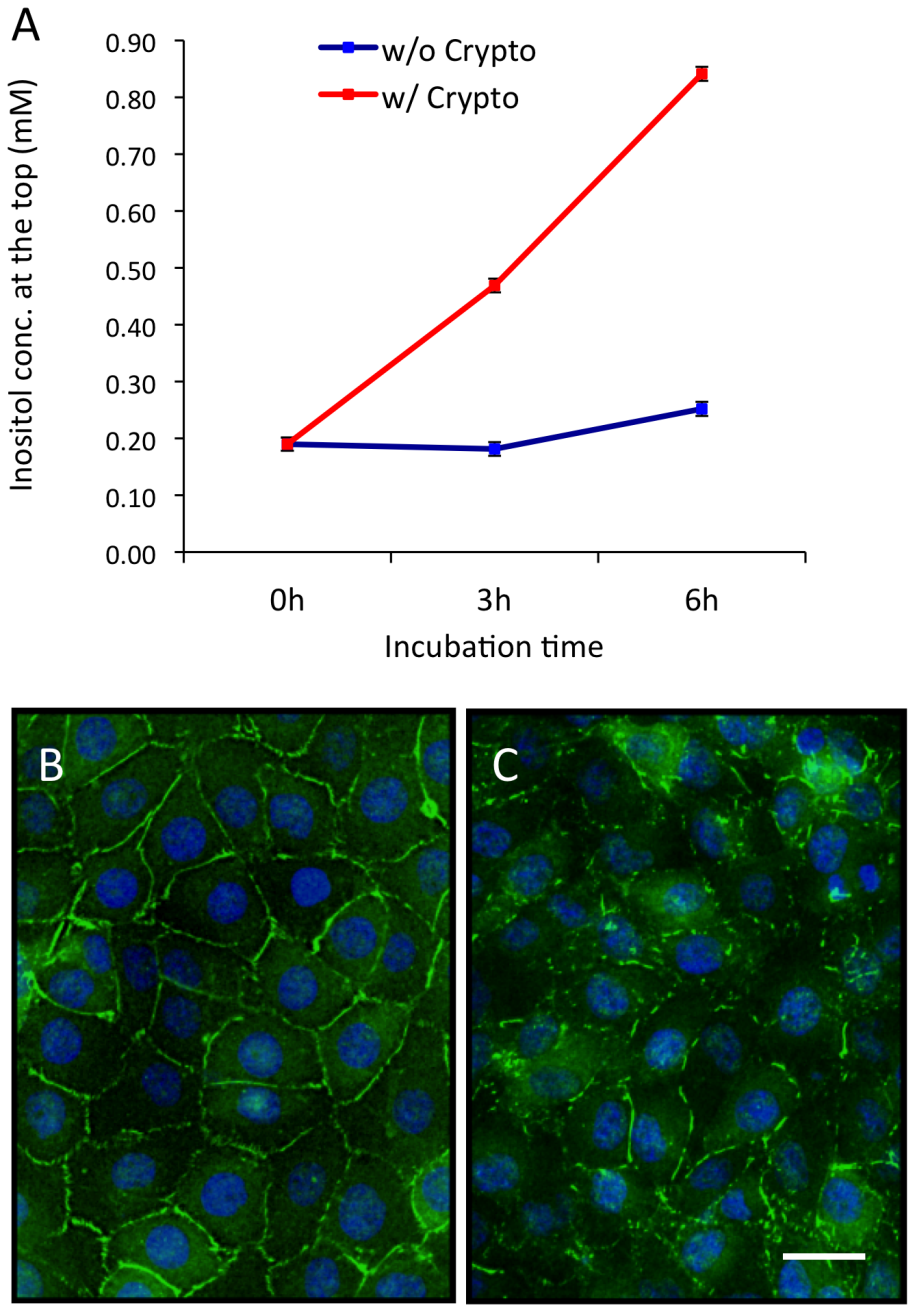
Determination of myo-inositol concentration in the top compartment. (A) The *in vitro* human BBB model was prepared with 1 mM inositol in the bottom compartment and incubated with or without 10^5^ *Cryptococcus* cells in the top compartment. After 3 and 6 hr incubations, the medium in the top compartment was collected and inositol concentration was determined. (B and C) Confluent HBMEC monolayers grown on the collagen-coated glass coverslips were incubated with medium only (B) or with the wild type H99 (C) for 1 hr. The cells were incubated with ZO-1 antibody, followed by Alexa488-conjugated secondary antibody. The coverslips were mounted on slides with Vectashield mounting solution containing DAPI and observed under a fluorescence microscope. ZO-1 (green) and nuclei (blue) are shown. Bar indicates 50 µm.

To understand how inositol in the bottom compartment affects *Cryptococcus* cells that are present in the top compartment, we measured the concentration of inositol in the top chamber by using an enzymatic method [46]. The medium alone contains around 0.18 mM inositol. In the absence of *Cryptococcus* in the top compartment, the addition of 1 mM inositol in the bottom compartment did not result in a measurable increase in the inositol levels in the top compartment after 3 hr incubation and only an increase to 0.25 mM inositol after 6 hr incubation, indicating that the HBMEC monolayer maintained high integrity and that inositol diffusion was very slow (Fig. 2A). In contrast, when 10^5^ *Cryptococcus* cells were added in the top compartment, the inositol level in the top compartment reached 0.47 mM after 3 hr and 0.84 mM after 6 hr (Fig. 2A). These results demonstrated that inositol can diffuse through the HBMEC monolayer from the bottom to the top compartment at a higher rate in the presence of fungal cells in the top compartment, possibly due to increased inositol permeability caused by *Cryptococcus*–HBMEC interactions.

Modification of tight junctions during *Cryptococcus* transmigration has been reported previously, which might contribute to the increased inositol permeability [40]. To test this hypothesis, we examined the integrity of tight junctions in response to *Cryptococcus* infection by immunofluorescence microscopy. Zona Occludens-1 (ZO-1) is a member of the tight junction protein complex and is widely used as a marker to determine the location of tight junctions [45]. The untreated HBMEC monolayer displayed continuous lining of ZO-1 staining pattern, suggesting intact tight junctions (Fig. 2B). However, *Cryptococcus* treatment induced dislocation of the ZO-1, results in the discontinued and scattered staining pattern between neighboring cells (Fig. 2C). These results provide evidence that the HBMEC-*Cryptococcus* interaction leads to the modification of tight junctions, which may contribute to the increased inositol permeability without causing major damage in the integrity of the HBMEC monolayer. To investigate whether such modification leads to the alteration of the integrity of the HBMEC monolayer, we also measured the transendothelial electrical resistance (TEER) of the monolayer. Our results showed a similar TEER readout in the absence or presence of yeast cells in the upper chamber (Fig. S1), suggesting there was no major change to the integrity of the monolayer, which is consistent with previous studies [25], [47].

### Inositol transporter genes of *C. neoformans* are required for its traversal across the HBMEC monolayer

**Figure 3:**
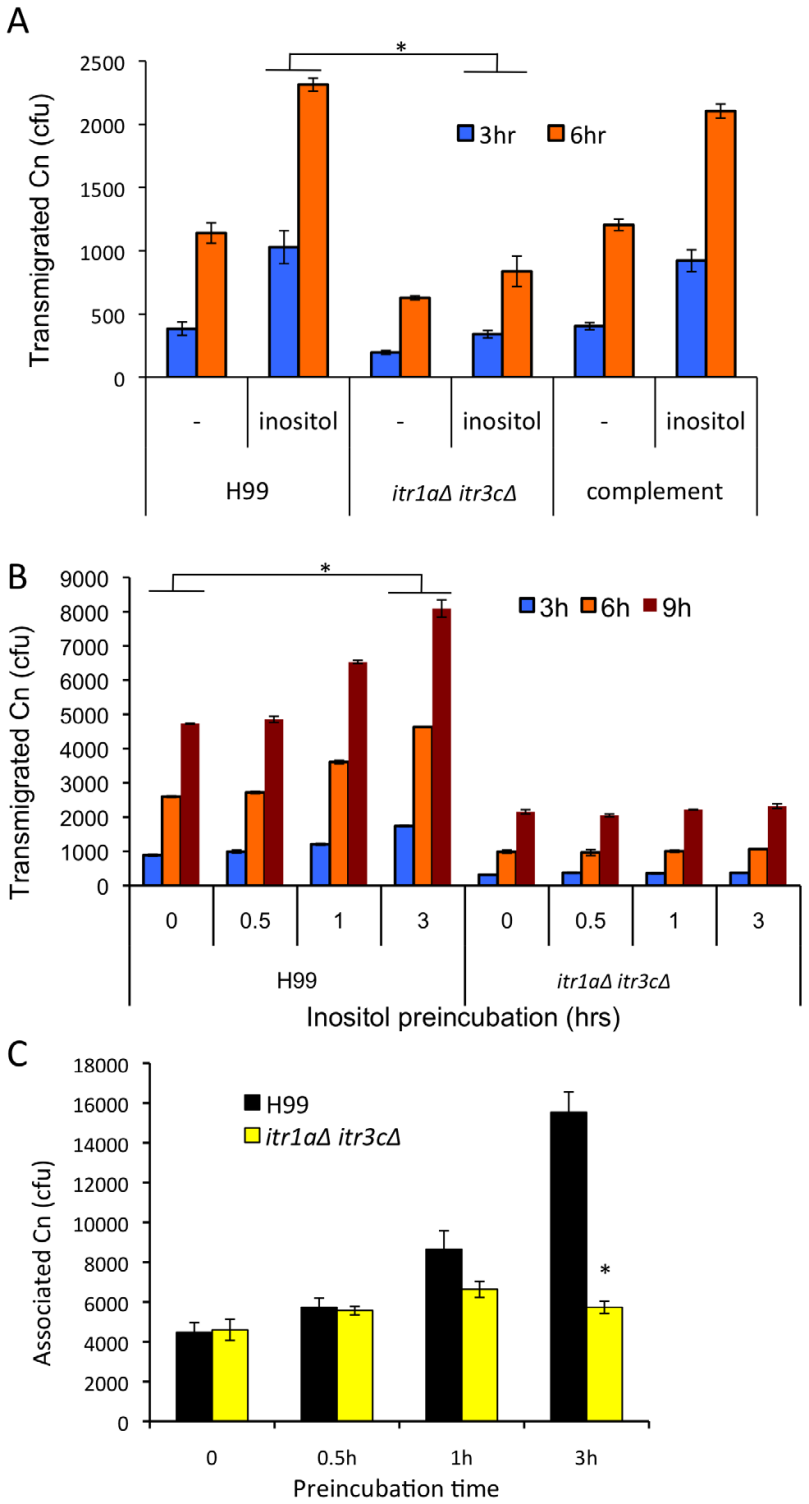
*ITR* genes are required for inositol-enhanced cryptococcal adhesion and transmigration across the BBB. (A) Transmigration assays were performed with H99, the *itr1aΔ itr3cΔ* mutant, and its complemented strain in the absence or presence of 1 mM inositol in the bottom compartment. (B and C) H99 and the *itr1aΔ itr3cΔ* mutant were pre-incubated with 1 mM inositol for the indicated times and their abilities to associate with the HBMEC monolayer and to transmigrate were determined by transmigration (B) and association (C) assays, respectively. Pre-incubated *Cryptococcus* cells were washed three times with the experiment medium to remove any residual inositol before assays. These assays were repeated three times independently. *Cn*: *Cryptococcus neoformans*. “*” indicates statistical significance (P<0.001).

Our previous studies demonstrated that *C. neoformans* has an unusually large inositol transporter (*ITR)* gene family, and established that *ITR*s were required for the full virulence of *Cryptococcus* in *in vivo* murine models [11], [22], [23]. Among them, Itr1a and Itr3c are two major *ITR*s for inositol uptake and fungal virulence [22]. To determine whether the attenuated brain infection of *ITR* gene deletion mutants was due to their defective ability to cross the BBB, we examined the transmigration ability of *C. neoformans itr1aΔ itr3cΔ* double mutants lacking these two major inositol transporter genes in our *in vitro* BBB model. In the absence of additional inositol, transmigration of the *itr1aΔ itr3cΔ* double mutant was decreased by 50% at 3 and 6 hr incubation periods compared to the wild type, indicating that *ITR* genes are required for cryptococcal crossing of the BBB. With inositol treatment, cryptococcal traversal was enhanced regardless of presence of *ITR* genes; however, a more significant defect of transmigration ability of *itr1aΔ itr3cΔ* mutants was evident. The number of transmigrated wild type H99 strain increased by 2.7 and 2-fold in the presence of inositol at 3 and 6 hr incubation, respectively, compared to transmigration in the absence of added inositol. In contrast, the *itr1aΔ itr3cΔ* double mutant exhibited approximately 1.7 and 1.3-fold increased transmigration rates after 3 and 6 hr incubation, respectively, with the result that the number of transmigrated *itr* double mutant cells was a third of that of the wild type strain H99 (Fig. 3A). The reduction in transmigration ability of the *itr1aΔ itr3cΔ* double mutant was fully restored by complementation.

Because inositol can be used as the carbon source for growth, there is a logistic concern that fungal cells with intact *ITR*s could grow better in medium with additional inositol. To address the concern, we compared the growth rate of wild type and the *itr1aΔ itr3cΔ* double mutant in the medium with or without addition of inositol. The results showed that all tested strains have similar growth rates regardless of the presence of inositol or *ITR* genes (Fig. 1C). Thus, the data are consistent with the interpretation that Itr1a and Itr3c play an important role in responding to inositol availability and contribute to cryptococcal traversal across the HBMEC monolayer.

We next pre-incubated *Cryptococcus* cells with 1 mM inositol (0.5, 1 and 3 hr), and then removed inositol by thorough washing with PBS before assessing transmigration (Fig. 3B). *Cryptococcus* cells pre-incubated for 30 min showed comparable transmigration to the untreated control, whereas longer pre-incubations of 1 or 3 hr enhanced the number of transmigrated fungal cells by 20% and 40%, respectively. However, similar enhancement in fungal transmigration was not detected with the *itr1aΔ itr3cΔ* double mutants following inositol pre-incubation (Fig. 3B). Association assays were then carried out with fungal cells pre-incubated with inositol to compare the ability of the wild type strain and the *itr1aΔ itr3cΔ* double mutant to associate with the HBMEC. The number of associated H99 cells was not changed after 30 min pre-incubation but was significantly increased after 3 hr compared to that of the untreated control (Fig. 3C). *Cryptococcus* cells pre-incubated for 1 hr exhibited a modest increase. Unlike the H99 wild type strain, the association rate of the *itr1aΔ itr3cΔ* double mutants was not changed by inositol pre-incubation (Fig. 3C), which is similar to the transmigration result shown in Fig. 3B. These results demonstrate that inositol uptake and utilization are required for efficient association and transmigration of cryptococcal cells. Our results also suggest that inositol uptake by *Cryptococcus* cells may lead to modification of the surface of *Cryptococcus* cells to enhance its association with and subsequent transmigration across the HBMEC monolayer.

### Involvement of *ITR*s in BBB crossing *in vivo*

**Figure 4:**
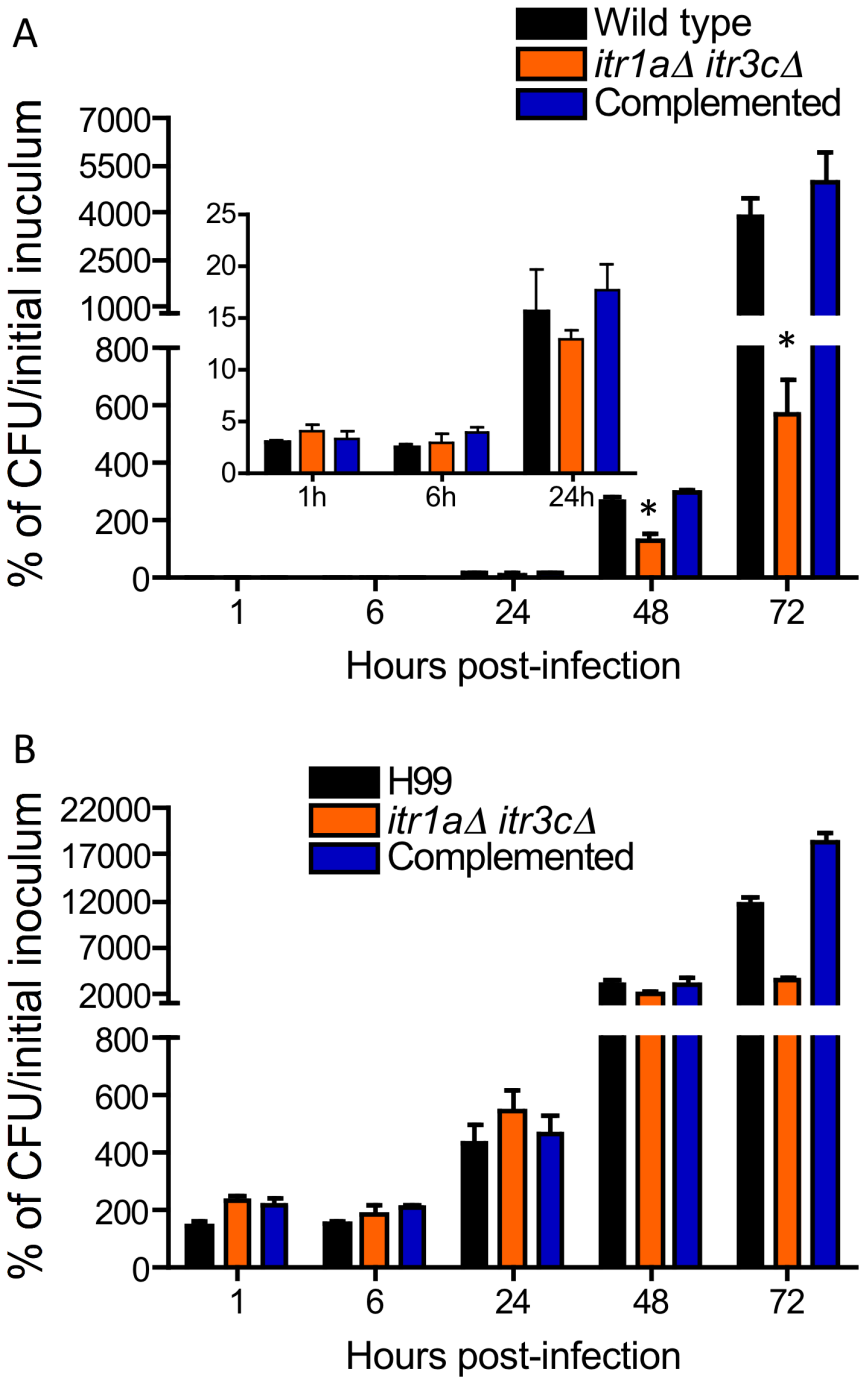
*ITR*s are required for traversal crossing of the BBB in a mouse intravenous model. CFUs were recovered from either the brain (A) and the lung (B) of mice injected with H99, the *itr1aΔ itr3cΔ* double mutant or its complemented strain at indicated times post-inoculation. CFU values are expressed as percentages of the initial inoculum. Error bars indicate standard deviations. “*” indicates statistical significance (P<0.001).

To further extend the results obtained from the *in vitro* human BBB model, we assessed infection with the *itr1aΔ itr3cΔ* double mutant in a murine model via intravenous injection. Infected mice were sacrificed at 1, 6, 24, 48, or 72 hr post-inoculation; brains and lungs were isolated and yeast CFUs were determined. Our results demonstrated that there was a significant difference in fungal burden in the brain between mice infected by wild type H99 or the *itr1aΔ itr3cΔ* double mutant after 24 hr post-infection. However, there was no significant difference in CFU at earlier time points (Fig. 4A). On the other hand, the fungal burden was similar in lungs infected either by wild type or the mutant at all time points except 72 hr (Fig. 4B). Our results thus demonstrate that inositol transporters Itr1a and Itr3c are required for fungal cells to either cross the BBB or grow in the brain after transmigration. It has been reported that although fungal crossing of the BBB occurred quite early after inoculation, the rate of traversal of *Cryptococcus* cells across the BBB increases dramatically 24 hr post-injection *via* tail vein [48]. We hypothesize there is a threshold effect with respect to time or number of cryptococcal cells accumulating on the endothelial monolayer before they can effectively penetrate the barrier. Fungal burdens at 72 hr post-injection were reduced in both brains and lungs infected by the mutant. However, the reduction is much greater in the brain (7-fold) compared to the reduction in the infected lung (4-fold) (compare 72 hr time point in Fig. 4). To investigate whether the mutant has a defect in survival in macrophage, we performed *Cryptococcus*-macrophage interaction assays using the macrophage-like cell line J774. Our results showed that wild type and the double mutant had similar response to phagocytosis and macrophage killing (Fig. S2).

**Figure 5:**
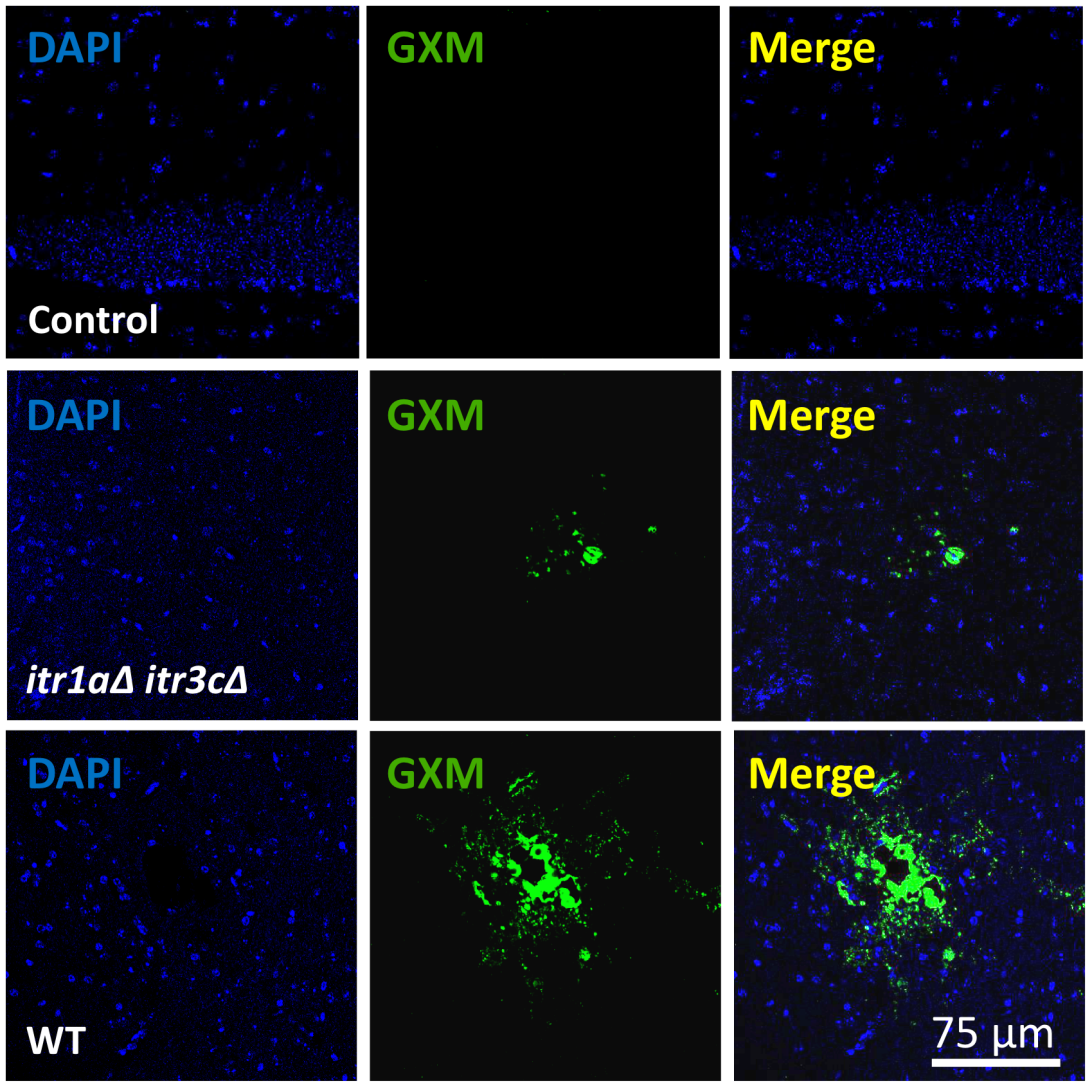
*Cryptococcus* cells penetrate into the brain by a mechanism involving fungal inositol transporters. Confocal analyses were performed using tissue sections obtained from mice that were either uninfected, or infected with wild type H99 (WT) or the *itr1aΔ itr3cΔ* mutant for 48 hr. Lesions were identified by the high accumulation of GXM within the CNS parenchyma (*Cn*, green channel) and DAPI staining (blue channel) to observe the nuclei in the cells. Animal brains infected with the mutant have less and smaller lesions. Bar indicates 75 µm.

To further understand the brain fungal infection at early time points post inoculation, we examined the presence of *Cryptococcus* within the CNS. To perform these studies, animals were infected with H99 or the *itr1aΔ itr3cΔ* double mutant for 48 hr before they were sacrificed to obtain the brain. Staining and subsequent confocal microscopy of 30–50 µm brain tissue sections was performed for polysaccharide glucuronoxylomannan (GXM) antibody to visualize the transmigration of cryptococcal cells into the CNS parenchyma. Our results showed that brains infected with H99 resulted in cryptococcal cells invasion of the CNS (5 to 7 lesions for each brain, especially in the cortex area). Transmigration of *Cryptococcus* cells was associated with large lesions (Fig. 5). Animals infected with the *itr1aΔ itr3cΔ* mutant present fewer lesions and cryptococcal cells within the CNS (Fig. 5, an average of 2 lesions by brain analyzed). The outcomes from fungal CFU counts and from immunofluorescent staining further support our hypothesis that fungal *ITR*s play a positive role in *Cryptococcus* traversal of the BBB in CNS infection. All these results are consistent with our CFU counts of infected brains (Fig. 4A).

### Itr1a and Itr3c are not required for fungal growth in infected brains

**Figure 6:**
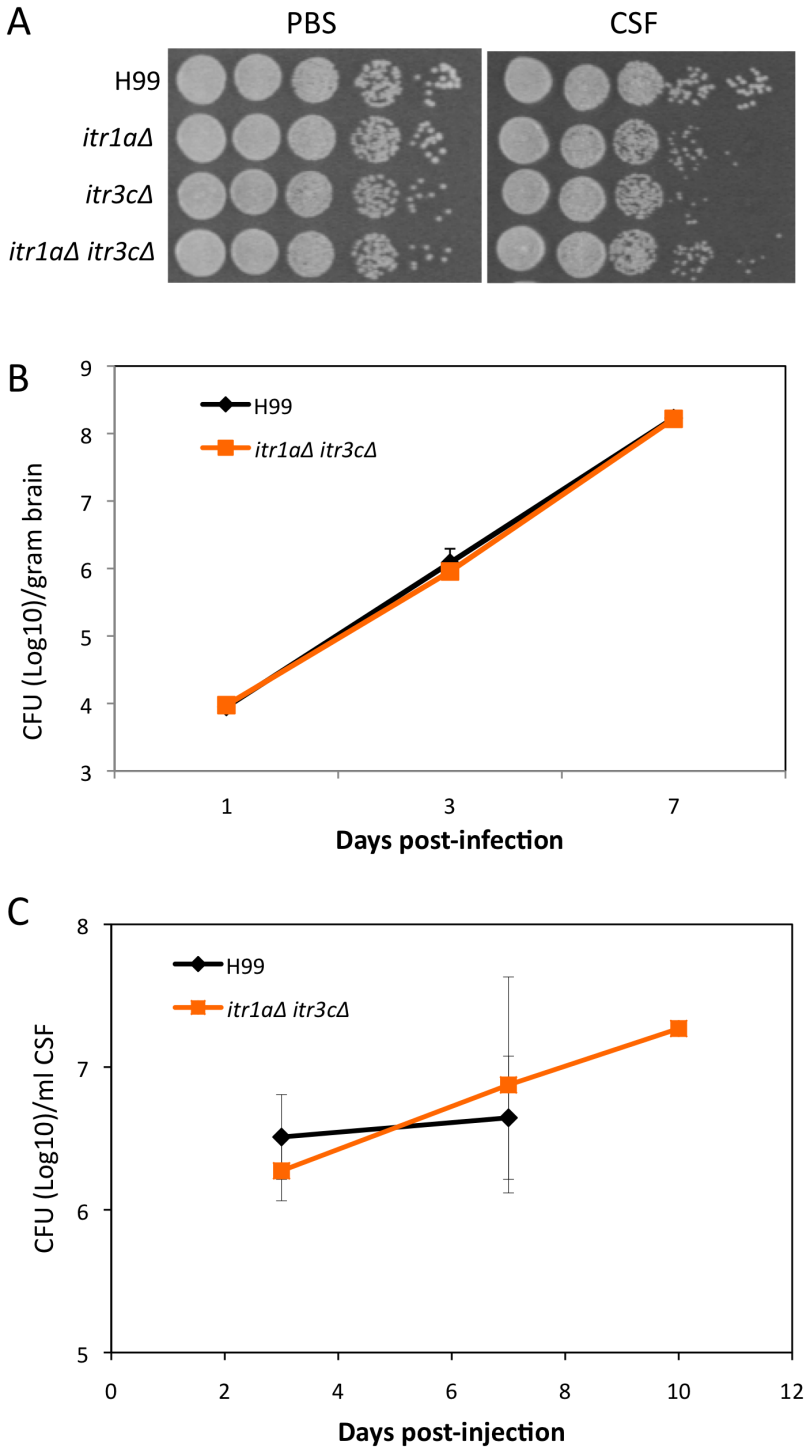
The *itr1aΔ itr3cΔ* double mutant does not exhibit a growth defect in cerebral spinal fluid (CSF). (A) Growth assays on plates using serial 10× dilutions were performed for H99, the *itr1aΔ* mutant, the *itr3cΔ* mutant, and the *itr1aΔ itr3cΔ* double mutant after treatment with either PBS buffer or purified human CSF. The results were photographed after 48 hr incubation. (B and C) Growth levels of H99 and the *itr1aΔ itr3cΔ* double mutant were compared by measuring the CFU of each strain isolated from total mouse brains (B) or CSF of rabbits (C) over time. Mice and Rabbits were infected intrathecally with 500 CFU and 10^8^ CFU of each strain, respectively. Fungal burden in infected mice were assess 1, 3, and 7 days post-inoculation, while fungal burden in rabbits was assessed 3, 7, and 10 days post-inoculation. The CFU data (10 days) for H99-infected rabbits was not shown since all three rabbits were dead less than 10 days post-infection. Error bars indicate standard deviations.

To address the possibility that the double mutant may grow slower once inside the brain, thereby leading to lower CFU recovery, we tested the growth of the *itr1aΔ itr3cΔ* mutant on an *in vitro* cerebral spinal fluid (CSF) medium that has been successfully used to identify strains with a growth defect in the CNS compartment [49]. There was no significant growth defect exhibited by the *itrΔ* single and double mutants on CSF medium (Fig. 6A).

To further confirm the *in vitro* growth results on CSF medium, we tested the *itr1aΔ itr3cΔ* double mutant in a murine intracerebral injection model of cryptococcosis. CFU from mouse brains were measured at 1, 3, and 7 days post-injection. Our results showed that at all three time points, brains (n = 4) infected by either the wild type H99 or the double mutant contain similar amount of fungal cells, indicating that there is no growth difference *in vivo* during brain infection (Fig. 6B).

We also compared the *in vivo* growth of wild type and the double mutant in a rabbit CSF model of cryptococcosis via intrathecal inoculation. This model allows us to measure the yeast CFUs from the same animal at different time points post-infection. CFU from the rabbit subarachnoid space were measured 3, 7, and 10 days post-infection. All rabbits (n = 3) infected by H99 were dead before 10 days, while one rabbit infected by the mutant remained alive at 10 days post-inoculation. Overall, these results showed that comparable numbers of CFUs were isolated from rabbits infected by either wild type or the mutant strain (Fig. 6C). This result further confirms that the defect in BBB traversal is the main reason for the virulence attenuation of the *itr1aΔ itr3cΔ* mutant shown in the murine tail vein injection model (Fig. 4A).

### *Cryptococcus* phospholipid composition is related to inositol treatment

**Figure 7:**
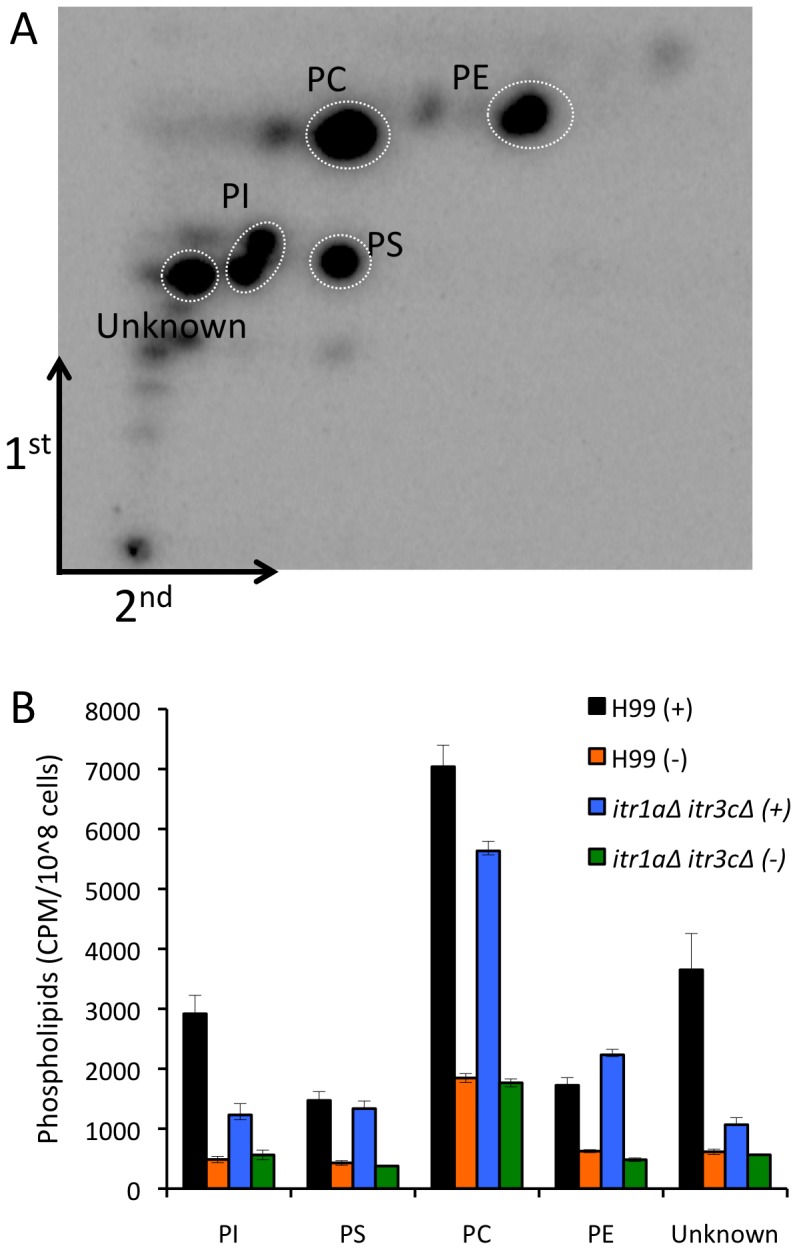
Inositol treatment changes phospholipid composition. Major phospholipids were examined by 2-dimensional thin layer chromatographic (2D-TLC) separation. H99 and the *itr1aΔ itr3cΔ* double mutant were steady-state labeled with ^32^Pi and grown on SD medium without or with 5 mM inositol for 20 hrs before cells were collected for lipid extraction. (A) A representative image of 2D-TLC results of H99 treated with inositol is shown. Five phospholipid species were labeled. (B) Quantification of phospholipid composition of *Cryptococcus* wild type H99 and the *itr1aΔ itr3cΔ* double mutant without or with inositol treatment. Abbreviations: PI, phosphatidylinositol; PC, phosphatidylcholine; PS, phosphatidylserine; PE, phosphatidylethanolamine; Unknown, one unidentified lipid species. Error bars indicate standard deviations.

Inositol is a precursor for the production of phospholipids, which are essential for cellular functions in eukaryotes. Because large amounts of inositol diffused from the bottom compartment into the top in our *in vitro* system, *Cryptococcus* can utilize the inositol available in the medium for its cellular functions. One possible explanation for the inositol effect on *Cryptococcus* transmigration is a change in phospholipid composition. To interrogate this hypothesis, we performed 2-dimensional thin layer chromatography assays (2D-TLCs) to evaluate the production of phospholipids in *Cryptococcus*. Our results showed that when yeast cells were grown on synthetic medium containing 5 mM inositol, the same phospholipid species were present in both H99 and the *itr1aΔ itr3cΔ* double mutant, but the production of phosphatidylinositol (PI) was two-fold lower in the mutant strain than in the wild type. Production of one unidentified lipid species was also significantly reduced in the mutant strain with inositol treatment (Fig. 7). Production of other major phospholipid species, e.g. phosphatidylcholine (PC), phosphatidylserine (PS), and phosphatidylethanolamine (PE), were similar between these two strains (Fig. 7). Hence, phospholipid composition, especially the difference in PI composition, could play a role in the dramatic reduction of transmigration in the double mutant. Furthermore, when we compared the phospholipids in H99 treated or not treated with inositol, we observed a dramatic difference in overall phospholipid levels, and addition of inositol significantly induced the production of all detected phospholipid species (Fig. 7). These results indicate that inositol plays a significant role in fungal phospholipid production, and could be part of the explanation for the inositol effect on *Cryptococcus* interaction with the HBMEC monolayer.

### Genes involved in inositol catabolism are highly upregulated by inositol incubation

To further understand how *Cryptococcus* cells respond to inositol, we analyzed transcriptional profiles of *Cryptococcus* that were treated or not treated with inositol to identify genes that are regulated by inositol. Cryptococcal cells were treated with 5 mM inositol for 24 hrs. Total RNA was prepared for hybridization with *Cryptococcus* 70-mer whole genome array chips. Our results showed that roughly 50 genes were significantly upregulated (>2 fold) (Table 1; Fig. S3), which is similar to the number of genes upregulated during mating under inositol induction conditions [23], while more than 300 genes were significantly downregulated (>2 fold) (Table 2). The complete list of genes with greater than two-fold change is shown in Table S1. Among upregulated genes, inositol oxygenase and beta-glucuronidase homologs were highly induced by inositol treatment (Table 1). Quantitative RT-PCR analyses were performed for six genes to confirm the microarray results (Fig. S3). The results demonstrated that the expression of two (myo)-inositol oxygenase genes (*MIO1* and *MIO2*), as well as two beta-glucuronidase genes (*CBG1* and *CBG2*) were indeed highly upregulated.

**Table 1:** Genes upregulated by inositol.

Protein ID		Description	Average fold changes
JEC21 ID	H99 ID		
Metabolism			
CN01750	CNAG_04394	Beta-glucuronidase (Cbg1)	33.11
CNF03950	CNAG_06623	Inositol oxygenase (Mio1)	32.63
CNF04890	CNAG_06530	Inositol 2-dehydrogenase (Idh1)	5.45
CNI01540	CNAG_04374	Beta-glucuronidase (Cbg2)	3.65
CNM02480	CNAG_06247	Glycosyl transferase	2.34
CNI00130	None	Polyadenylation factor	2.33
CNG03010	CNAG_03277	Inositol oxygenase (Mio2)	2.21
Signal transduction			
CNG04220	CNAG_03143	Heat shock protein	2.38
CNL05090	CNAG_00030	Fungal specific transcription factor	2.25
CNF03740	CNAG_06642	Phosphatidylinositol 3-kinase	2.22
CNM02200	CNAG_06220	Allergen	2.24
Inositol transporters			
CND00070	CNAG_00867	Itr3a	1.99
CNA00880	CNAG_00097	Itr1	1.66
CNH03060	CNAG_05667	Itr3b1	1.44
CNG02980	CNAG_03357	Itr2a	1.26
CNB05060	CNAG_04024	Itr5	1.08
CNH00990	CNAG_05381	Itr3c	1.23
CNH02990	CNAG_05662	Itr4	0.95
CND00020	CNAG_00864	Itr2	0.83
Proteins with unknown function			
CNL03670	CNAG_05261	*Cryptococcus* specific protein, unknown (Isp1)	2.42
CNJ03330	CNAG_07840	*Cryptococcus* specific protein, unknown (Isp2)	2.42
CNK02130	CNAG_01873	Fungal specific protein, unknown	2.27
CNJ03390	CNAG_04919	Unknown	2.18

**Table 2:** Genes downregulated by inositol.

Protein ID		Description	Average fold changes
JEC21 ID	H99 ID		
Metabolism			
CNH03280	CNAG_05303	Isocitrate lyase	−5.27
CNA00470	CNAG_00057	Fructose-1,6-bisphosphatase	−5.02
CNA04370	CNAG_00457	Glutamine synthetase	−4.41
CNF03900	CNAG_06628	Aldehyde dehydrogenase	−4.31
CNC02480	CNAG_01745	sn-Glycerol-3-phosphate dehydrogenase	−3.52
CNF04450	CNAG_07660	Pyruvate dehydrogenase	−3.51
CNA01050	CNAG_00115	Sorbitol dehydrogenase	−3.33
‘CNJ00950’	CNAG_04659	Pyruvate decarboxylase	−3.23
CNG00600	CNAG_07745	Mannitol-1-phosphate dehydrogenase	−3.00
CNC06440	CNAG_01539	Inositol-1-phosphate synthase (Ino1)	−2.79
CNE01560	CNAG_02399	Glutathione reductase	−2.72
CND00180	CNAG_00879	Glutamate dehydrogenase	−2.63
CNA04610	CNAG_07363	Isocitrate dehydrogenase	−2.59
CNL04840	CNAG_05138	Exo-beta-1,3-glucanase	−2.54
CNC02410	CNAG_01737	Methyl sterol oxidase	−2.54
CNB00990	CNAG_03596	2-Oxoglutarate_dehydrogenase_complex	−2.53
CNE02710	CNAG_07639	Triacylglycerol lipase	−2.50
Signal transduction			
CNA01180	CNAG_00130	Serine/threonine protein kinase	−5.11
CNB05690	CNAG_04090	bZip transcription factor	−3.13
CNB01230	CNAG_03621	Cyclophilin A	−2.96
CNH00140	CNAG_05348	Small_GTPase_CDC42	−2.88
CNH00970	CNAG_05431	Transcription factor PacC-related	−2.76
CNE03320	CNAG_02209	SIK1	−2.43
CNB01720	CNAG_03673	Serine/threonine protein kinase	−2.39
CNG00140	CNAG_03591	Secretory pathway gdp dissociation inhibitor	−2.30
CNA01820	CNAG_00193	Transcription factor ScGAT	−2.25
CNC04340	CNAG_02817	Rab/GTPase	−2.17
CNB04890	CNAG_04005	Negative regulator of differentiation 1	−2.12
CNH00600	CNAG_05392	ROK1 related	−2.08
CNI03350'	CNAG_04197	Yak1 homolog	−2.06
Membrane proteins			
CNE02570	CNAG_02288	Succinate/fumarate mitochondrial transporter	−3.22
CNA07060	CNAG_00727	Cation transporter-like protein	−2.62
CNB04770	CNAG_03991	GPCR	−2.46
CNA00570	CNAG_00066	Membrane protein	−2.39
CNL04850	CNAG_05137	Putative KDEL receptor	−2.14
CNH00490	CNAG_07874	Sugar transporter	−2.06
CNC00290	CNAG_03060	Multiple drug resistance protein	−2.04

*Cryptococcus* can use inositol as a carbon source. Conversion of inositol to glucuronic acid by inositol oxygenases (*MIO*s) is the first step of the only known pathway for inositol catabolism: the oxygenase controls the utilization of inositol as an energy source [50], [51]. *C. neoformans* has three *MIO* homologues (CNAG_06623, CNAG_03277, and CNAG_05316), which is another unusual feature that may be related to inositol function in this fungus. Multiple copies of the *MIO* gene is unique to *Cryptococcus* among the animal and fungal kingdoms [51]. Beta-glucuronidase is a member of the glycosidase family that catalyzes the breakdown of complex carbohydrates by releasing the glucuronic acid residues from polysaccharides [52]. In addition, a few inositol transporters are upregulated by inositol, while the gene encoding inositol 1-phosphate synthase (*INO1*) is significantly downregulated, suggesting, as we expected, that these two inositol acquisition pathways are regulated by inositol (Table 1 & 2).

### Inositol treatment induces the production of hyaluronic acid in *C. neoformans*

**Figure 8:**
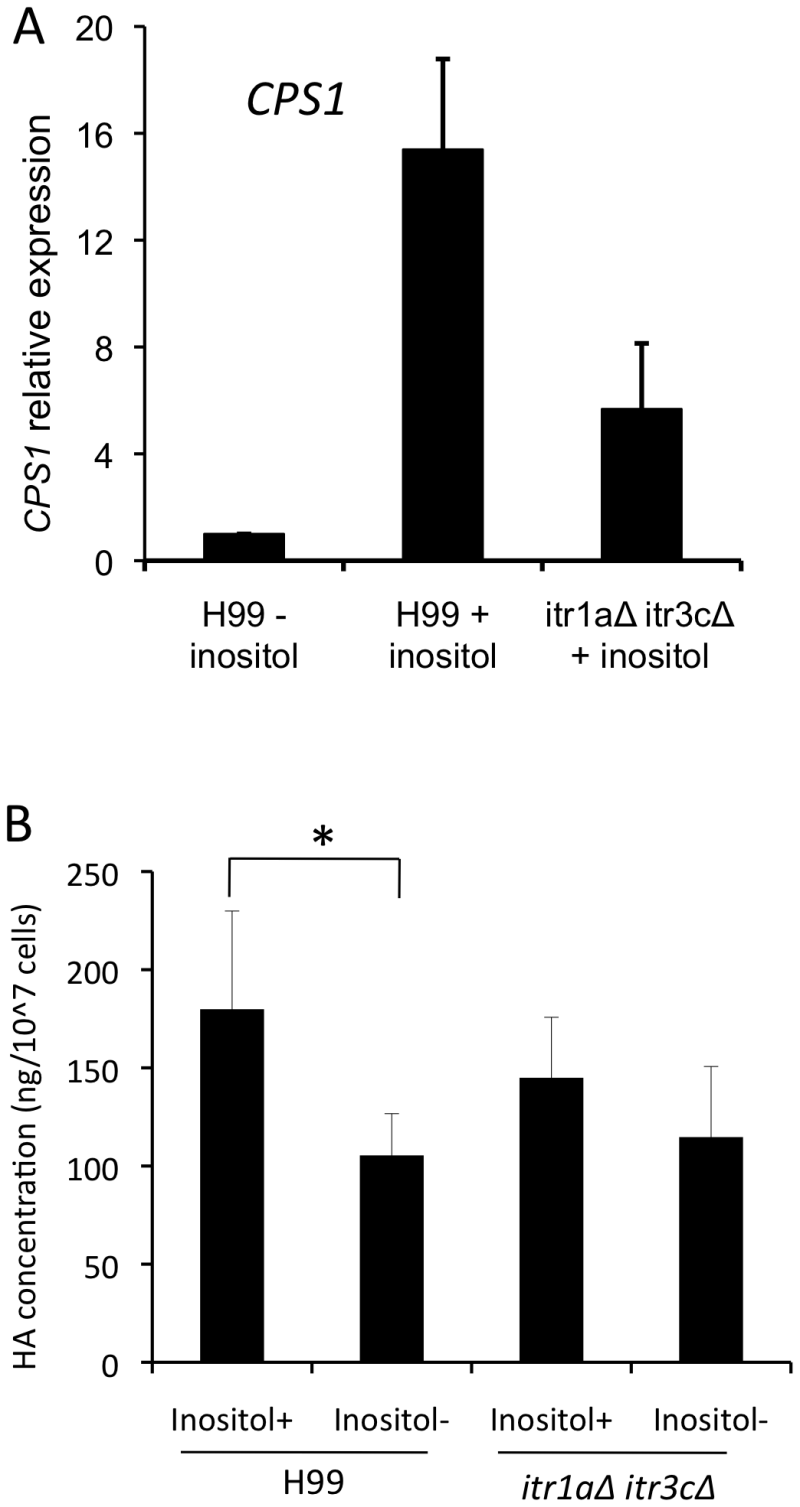
Inositol plays a positive role in hyaluronic acid production. The expression of hyaluronic acid synthase, *CPS1*, was determined by qRT-PCR (A), and hyaluronic acid production was measured using the HA-ELISA kit (Corgenix, Colorado) (B) for strains including wild type H99 and the *itr1aΔ itr3cΔ* double mutant that are either treated by 5 mM inositol (+) for 24 hrs or without treatment (−). The results shown are from at least three independent replicates. Error bars indicate the standard deviations. “*” indicates statistical significance (P<0.001).

Cps1 is identified as the hyaluronic acid synthase in *Cryptococcus* [53], [54]. Hyaluronic acid has been reported to be a *Cryptococcus* ligand that can bind to the CD44 glycoprotein in HBMECs [55]. Using a quantitative RT-PCR analysis, we also detected high induction of *CPS1* gene expression following treatment with inositol (Fig. 8A). The overproduction of hyaluronic acid likely increases the association and transmigration of *Cryptococcus*. Therefore, we measured hyaluronic acid production using a hyaluronic acid ELISA kit (Corgenix, Colorado, AZ). The results showed that the production of hyaluronic acid in wild type H99 was significantly increased (P<0.001) when the medium contained 1 mM inositol, confirming that inositol regulates hyaluronic acid production in *C. neoformans*, leading to an increased rate of fungal association and transmigration. In addition, the *itr1aΔ itr3cΔ* double mutant also produced a reduced level of hyaluronic acid compared to H99 in the presence or absence of inositol in the medium, but the reduction is less significant (P = 0.12) (Fig. 8B). This outcome suggests that inositol regulates the production of hyaluronic acid, but additional mechanisms may also be involved in fungal transmigration.

### Treatment with an inositol transporter inhibitor blocks *Cryptococcus* transmigration

**Figure 9:**
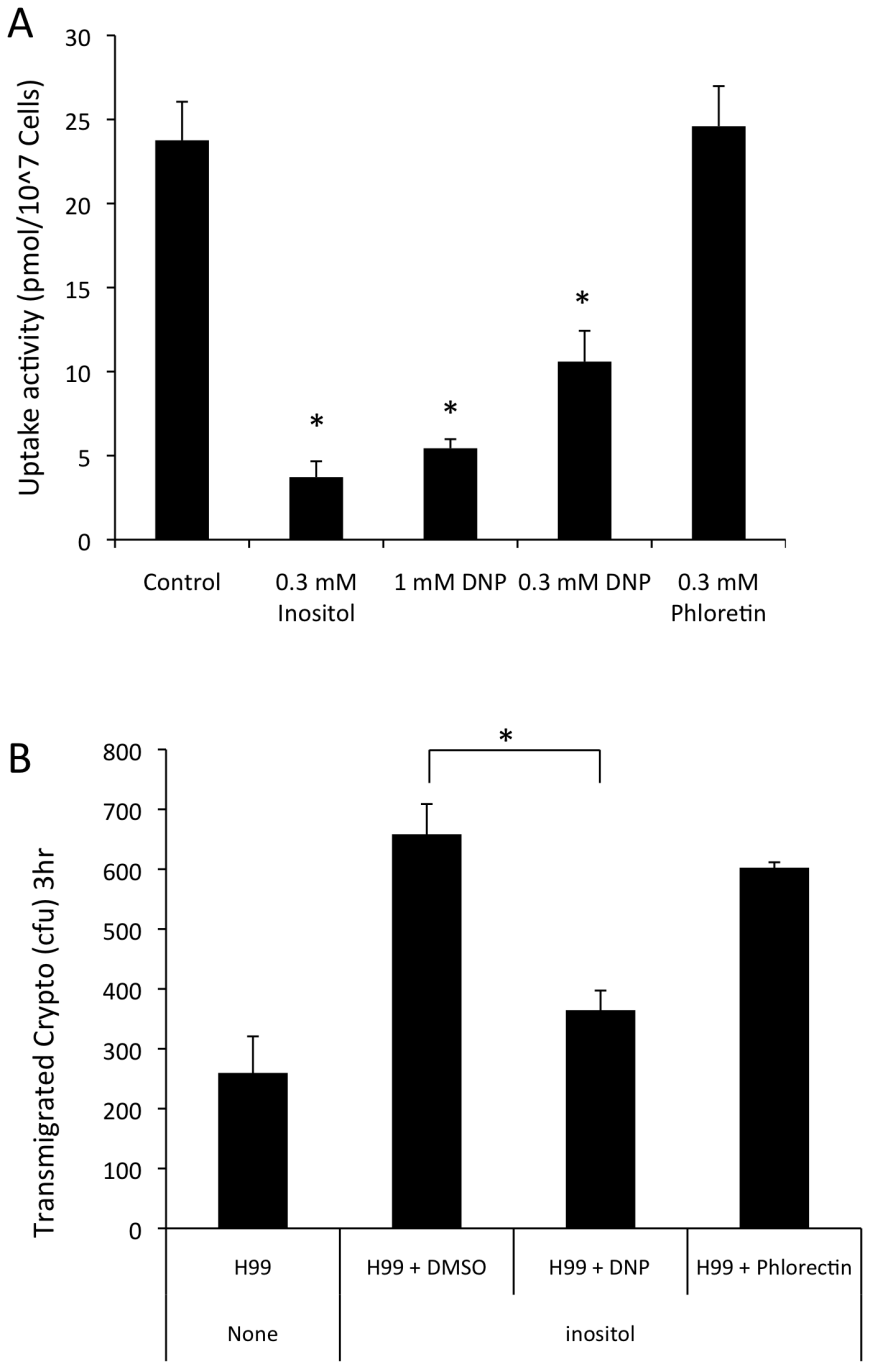
Blockage of inositol uptake inhibits *Cryptococcus* transmigration of the HBMEC monolayer. (A) Inositol uptake assays for H99 strain were performed with the ^3^H-labeled myo-inositol in the presence of cold myo-inositol or inositol transporter inhibitors dinitrophenol (DNP) and phloretin. (B) *C. neoformans* (H99) was incubated with either vehicle (DMSO), DNP (0.3 mM) or Phloretin (0.3 mM). After 30 min incubation, H99 was washed with experiment medium to remove inhibitors and added to Transwells. Transmigration assays were performed for 3 hr and the number of H99 crossing the HBMEC monolayers were determined by counting CFU. Each assay was set up in triplicate and independently performed twice. “*” indicates statistical significance (P<0.001).

Fungal inositol transporters are proton-dependent, which is different kinetically and pharmacologically from the sodium-dependent myo-inositol transporters (*SMIT*s) in mammalian cells [56]. Thus, fungal inositol transporters have potential as attractive drug targets. Dinitrophenol (DNP) is a protonophore that dissipates transmembrane proton gradients and has been shown to effectively block inositol uptake in *Candida albicans* [56]. We treated *Cryptococcus* wild type strain H99 with DNP or with the human sodium-dependent inositol transport inhibitor phloretin before performing inositol uptake assays. The inositol uptake assays revealed that DNP produced a pronounced inhibitory effect on inositol import in *Cryptococcus*, while phloretin had no obvious effect (Fig. 9). Subsequently, DNP or phloretin pretreated H99 cells were used in fungal transmigration assays in the *in vitro* BBB model. The results demonstrated that the DNP pretreatment leads to a significant reduction in cryptococcal transmigration stimulated by the addition of inositol, compared to those treated with a vehicle (DMSO) or phloretin. These findings further confirm that cryptococcal uptake of host inositol is through proton-dependent inositol transporters, and is required for assisting *Cryptococcus* traversal across the HBMEC monolayer. Due to the nature of protonophore, DNP treatments may cause effects on cryptococcal cells in addition to inhibiting inositol uptake, such as effects on mitochondria function. To address the concern that DNP may alter the growth of cryptococcal cells, we compared the growth rates of H99 cells treated with or without either DNP or phloretin. The results showed that the different treatments did not affect growth curves (Fig. S4), indicating that the decreased cryptococcal transmigration was due to reduced inositol uptake in H99 rather than an effect on other fungal physiology. Taken together, these findings further confirm that host inositol plays a positive role in *Cryptococcus* traversal across the BBB.

## Discussion

The mechanisms for the frequent occurrence of Cryptococcal CNS infection remain unclear. In this study, we explored our hypothesis that the high abundance of inositol in human brain contributes to virulence of *Cryptococcus* and the development of cryptococcal meningitis. In our *in vitro* model of BBB using the HBMEC monolayer, we observed that inositol promotes an increase in *Cryptococcus* association with and transmigration through the HBMEC monolayer. This increase is dependent upon fungal inositol transporters (*ITR*s), demonstrated by the fact that mutation of two major *ITR*s, *ITR1A* and *ITR3C*, partially abolishes the stimulation of *Cryptococcus* transmigration by inositol. Our results indicate that inositol plays a role in the traversal of *Cryptococcus* across the BBB, and showed that fungal cells can respond to inositol availability in the brain. Because we measured the transmigration rate by counting yeast cell numbers in the bottom chamber in our *in vitro* model, one concern in interpreting our results is whether addition of inositol to the bottom chamber has an effect on the proliferation of yeast cells. We measured the proliferation rate of *Cryptococcus* cells in HBMEC medium with or without addition of inositol, and observed a similar growth rate in all strains tested, confirming that the difference in cell numbers in the bottom compartment reflected a difference in yeast transmigration. *Cryptococcus* can use inositol as a carbon source although it is not a preferred source; *Cryptococcus* grows very slowly on medium containing inositol as the sole carbon source. Glucose is a much better carbon source for *Cryptococcus*. The HBMEC medium is enriched in nutrients and contains sufficient glucose for optimal fungal growth, which would explain why inclusion of 1 mM inositol did not affect cell growth. In addition, the association assays demonstrate that inositol promotes the association of *Cryptococcus* with the HBMEC monolayer. Because a better association often leads to increased transmigration, this effect could explain the increase in transmigration. In addition, because inositol is highly abundant in human and animal brains, for example, the astrocytes that directly interact with the BBB contain over 8 mM inositol that can be rapidly released [38], [39], we believe the inositol level in the brain side of the BBB is high and the inositol concentrations we used in *in vitro* assays are physiologically relevant.

Our *in vivo* study using a murine model revealed a significant difference in numbers of CFUs recovered from mouse brains 24 hr post tail vein injection between mice infected by wild type versus the *itr1aΔ itr3cΔ* double mutant, confirming that the double mutant has a defect in brain infection. In the mutant, the observation of lower fungal burden in brains but not in lung during early infection suggests a defect in traversal across the BBB, consistent with our results from the *in vitro* system. We did observe a significant reduction in CFU in mutant-infected lungs 3 days post-inoculation, although the reduction was much less pronounced than in infected brains. Because the *itr1aΔ itr3cΔ* double mutant exhibits a moderate melanin defect [22], the reduced fungal burdens in the mutant infected lungs over time may be partially caused by reduced laccase activity, a known virulence factor [57]. However, these additional effects cannot account for the observed reduction in transmigration of cells carrying the double mutation, especially during early infection. Our confocal images of immunofluorescent staining further confirmed that *Cryptococcus* cells transmigrate into the brain by a mechanism that depend in part on inositol transporters, because strains lack of Itr1a and Itr3c have reduced fungal invasion in the brain.

The outcome of our animal studies on transmigration could be influenced by the impact of the CNS environment on yeast growth and survival after transmigration. To address this biological artifact on transmigration analysis, we examined growth and/or survival of H99 and the double mutant within relevant biological fluids. Both in human CSF and in animal models of cryptococcal meningitis there was no apparent influence of the inositol transporters on yeast growth or survival *in vivo*, supporting their early impact on transmigration rather than direct effect on CNS compartmental survival and growth. However, we could not completely rule out that there may be a growth difference in certain microenvironment during brain infection, such as certain parts of the brain parenchyma, which is bypassed in the intracerebral injection model. In addition, we have shown that the presence of inositol transporters did reduce survival of the host in a murine intracerebral infection model [22]. Therefore, Itr1a and Itr3c are necessary for full virulence at this CNS site of infection. In fact, our preliminary results from a RNA-SEQ analysis of infected brains indicated that the double mutant caused more active host defense response than wild type, a potential explanation of the virulence attenuation of the double mutant during the establishment of the CNS cryptococcosis (our unpublished data). Therefore, inositol utilization likely influences on fungal transmigration across the BBB as well as subsequent disease development in the CNS.

Furthermore, studies have shown that yeast cells cross the BBB early after infection, but that a dramatic increase occurs 24 hr post-injection [48]. Thus, a significant defect in the transmigration may not be detectable before 24 hr. We hypothesize that there is a threshold effect with respect to time or number of cryptococcal cells associated with brain microvascular endothelial cells before they can effectively penetrate the BBB *in vivo*. This is not without precedent; it has been reported that a bacteremia approaching 10^3^ cells per milliliter in bloodstream is a prerequisite for meningitis-causing *E. coli* K1 to cross the BBB [58], [59].

In the *in vitro* BBB model used in this study, inositol added in the bottom compartment can diffuse to the top compartment to form an inositol concentration gradient. Our analysis showed that there was a time-dependent increase of inositol concentration in the top compartment with the presence of *Cryptococcus*, while very little increase of inositol level at the top without incubation with *Cryptococcus*. Furthermore, *Cryptococcus* incubation induces the modulation of the tight junction, as shown by the ZO-1 dislocation. These results suggest that the *Cryptococcus*-HBMEC interaction is required to modulate the permeability of the HBMEC monolayer to inositol. It has been reported that *Cryptococcus* invasion causes a modification of tight junctions [40]. In addition, inositol uptake by the host cells may contribute to the inositol level increase in the top compartment, since host cells also have inositol transporters. Our studies have shown that inositol alone is able to stimulate HBMEC and its uptake by HBMEC enhances the *Cryptococcus*-mediated phosphorylation of host signaling proteins and the permeability of dextran across the HBMEC monolayer in the presence of *Cryptococci* (Kim et al., unpublished). Alternatively, it is also possible that *Cryptococcus* and HBMECs may release some amount of inositol into the medium during their interactions. However, despite the increase in inositol permeability and ZO-1 dislocation, TEER measurement of the HBMEC monolayer was not significantly changed during incubation with *Cryptococcus*, suggesting that inositol diffuses through the monolayer without causing major damage to the BBB, which is consistent with previous reports [25], [47]. Further characterization on the inositol effects on the *Cryptococcus*-mediated modulation of HBMEC barrier is required to precisely understand the mechanisms for inositol diffusion.

Because both Itr1a and Itr3c are major inositol transporters, *Cryptococcus* may be able to import inositol for its cellular function. It is possible that inositol uptake through inositol transporters modulates *Cryptococcus* cells to enhance their association with HBMEC and transmigration across the BBB. Our 2D-TLC analysis demonstrated that, in both the wild type and the *itr1aΔ itr3cΔ* double mutant, incubation with inositol significantly increases the amount of phospholipids produced (Fig. 7). Interestingly, among phospholipids, the amount of phosphoinositide (PI) detected after inositol treatment was much lower in the *itr1aΔ itr3cΔ* mutant than in the wild type. PI is a precursor in the production of inositol phospholipid and other downstream inositol metabolites that are essential for cellular function. Changes in PI production could profoundly affect *Cryptococcus* cellular signaling regulation and modification of cell surface dynamics. Phospholipids, including phosphatidylcholine (PC), have recently been identified to play a role in the capsule enlargement [60]. In our TLC results, PC production is highly induced by inositol treatment. The role of the capsule in *Cryptococcus* transmigration remains controversial in that some studies have demonstrated that capsule is involved [48], [61], while other reports have suggested a capsule-independent brain invasion process [26], [62]. Although we did not detect an obvious difference between the mutant and wild type in capsule size based on microscopy, it is possible that inositol may play a role in capsule structure or density, which may also influence cryptococcal transmigration and/or other functions during *Cryptococcus*-host interactions.

Microarray analysis also revealed that inositol treatment induces upregulation of genes related to inositol catabolism and metabolism. Converting inositol to glucuronic acid by inositol oxygenases is the first step of the only known inositol catabolism pathway [50]. *Cryptococcus* has three genes encoding oxygenase enzymes and the expression of two of them is highly induced by addition of inositol, suggesting that the addition of inositol stimulates the production of glucuronic acid. The upregulation of beta-glucuronidase genes by inositol may indicate that yeast cells have increased carbohydrate turnover, resulting in overproduction of polysaccharides due to inositol supplementation. Whether inositol-converted glucuronic acid can be used to produce UDP-glucuronic acid, as one substrate of the polysaccharide capsule, remains uncertain. Previous studies have shown that UDP-glucuronic acid in *C. neoformans* is exclusively produced by the glycolytic pathway from glucose [63], [64]. However, it remains a possibility that under certain culture conditions, such as when inositol is used as a sole carbon source, glucuronic acid can also be a precursor of UDP-glucuronic acid.

UDP-glucuronic acid is also one substrate for the synthesis of hyaluronic acid, a *Cryptococcus* ligand that interacts with CD44 on host cells during fungal invasion and transmigration of the BBB [36], [65]. Interestingly, we found that the gene encoding hyaluronic acid synthase, *CPS1*, is upregulated by inositol treatment and leads to the induction of hyaluronic acid production. The increased production of hyaluronic acid indicates that inositol may enhance the association between *Cryptococcus* and brain endothelial cells. Therefore, the changes induced in *Cryptococcus* through inositol uptake may play a positive role in fungal association as well as transmigration. The fact that the *itr1aΔ itr3cΔ* double mutant only showed a marginal reduction in hyaluronic acid production compared to wild type is consistent with results demonstrating that this mutant can invade the BBB and cause infection although less efficiently. Additional factors, such as the influence of inositol on the cellular lipid rafts, caveolin-1, cytoskeleton, etc., could also be in play to alter the transmigration. As well, additional redundant *ITR*s may contribute and/or partially compensate for losses in the double mutant background. A mutant strain lacking the whole *ITR* gene family would be valuable for understanding the complete role of *ITR*s. We are in the process of generating such a mutant strain.

In addition, inositol itself is an important signaling molecule that affects many biological functions. Inositol stimulates the long distance sodium uptake from root to leaf in plants [66], and many species of caterpillar can sense inositol on plants for feeding [67], [68]. *Cryptococcus* may sense the concentration gradient of inositol and promote its association with endothelial cells and increase the transcytosis in response to higher inositol concentration, a phenomenon similar to chemotropism. The role of inositol as a potential chemoattractant to stimulate *Cryptococcus* to associate and penetrate the BBB needs to be further investigated..

**Figure 10:**
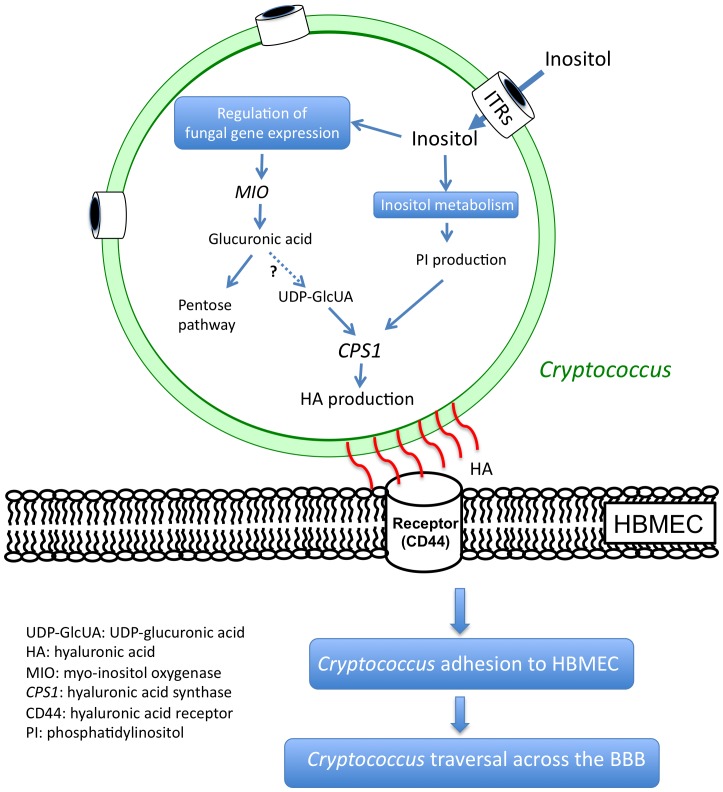
Current model of the role of myo-inositol in *Cryptococcus* traversal across the BBB. *Cryptococcus* cells sense the presence of inositol diffused from the brain, and take up inositol using inositol transporters. Inositol uptaken into *Cryptococcus* cells leads to the increased production of phosphatidylinositol (PI) and influences fungal gene expression including *CPS1* involved in the synthesis of hyaluronic acid (HA). Upregulation of HA production leads to enhance cryptococcal binding to human brain microvascular endothelial cells (HBMEC) and subsequently transmigration across the BBB to gain access into the CNS. Abbreviations: UDP-GlcUA, UDP-glucuronic acid; *MIO*, myo-inositol oxygenase; *CPS1*, hyaluronic acid synthase.

Overall, we have demonstrated that brain inositol affects fungal cells to promote the traversal across the BBB as presented in our model (Fig. 10). Cryptococcal cells disrupt tight junctions of the BBB allowing leakage of inositol from the brain to the bloodstream to generate an inositol concentration gradient; *Cryptococcus* senses the presence of inositol gradient via inositol sensors that remain to be identified, and takes up inositol using these inositol transporters. Inositol import leads to modifications in fungal physiology such as hyaluronic acid production. The inositol-mediated changes on fungal cells lead to enhanced yeast binding to and transmigration of the BBB, resulting in cryptococcal brain infection and disease development. This model suggests that there is a complex interaction between fungal cells and host cells that involves host inositol and fungal inositol transporters.

Because the transmigration and subsequent disease establishment in the brain is a continuous process that is difficult to separate definitely, we cannot rule out the possibility that in addition to its involvement in the fungal transmigration, inositol utilization could also play an important role in the establishment of cryptococcal meningitis in the CNS. In fact, this hypothesis is supported by our unpublished data on the difference in host immune response between brains infected by the wild type and the *itrΔ* mutant.

Despite the importance of host inositol, we do aware that inositol may be only one of multiple factors that affect the development of the CNS cryptococcosis. Additional factors and mechanisms likely also exist to stimulate the fungal cell transmigration and the establishment of fungal infection in the brain. In fact, several factors, such as capsule [48], [61], urease [30], [31], phospholipase B1 [32], [33], and host copper utilization [69], have been reported to play a role in cryptococcal dissemination and/or brain infection. Nevertheless, our discovery of the involvement of host inositol and fungal inositol transporters in the development of cryptococcal CNS infection leads to a better understanding of this complex host-pathogen interaction during the development of cryptococcal meningitis.

## Material and Methods

### Ethics statement

The animal studies conducted at Duke University and University of Medicine and Dentistry of New Jersey (UMDNJ) were in full compliance with all of the guidelines set forth by the Institutional Animal Care and Use Committee (IACUC) and in full compliance with the United States Animal Welfare Act (Public Law 98–198). The Duke and UMDNJ IACUCs approved all of the vertebrate studies. The studies were conducted in facilities accredited by the Association for Assessment and Accreditation of Laboratory Animal Care (AAALAC).

### Strains, media, and growth conditions

*C. neoformans* var. *grubii* (serotype A) H99 and its isogenic mutant strains (*itr1aΔ, itr3cΔ, itr1aΔ itr3cΔ*) have been previously described [22], [23]. *C. neoformans* var. *neoformans* (serotype D) strain B-3501 was kindly provided by Dr. Kyung J. Kwon-Chung (NIAID), whereas *Candida albicans* strain ATCC90028 was kindly provided by Dr. David Perlin (UMDNJ). Yeast cells were grown on YPD (1% yeast extract, 2% peptone, 2% glucose) agar plates and synthetic (SD) medium at 30°C and stored at 4°C until use. The Anti-GXM antibody 18B7 was kindly provided by Dr. Arturo Casadevall (Albert Einstein College of Medicine). Horseradish peroxidase-conjuagated anti-mouse and anti-rabbit antibodies were obtained from Invitrogen (Grand Island, NY). Dinitrophenol and inositol isomers were purchased from Sigma-Aldrich (St. Louis, MO).

### Human brain microvascular endothelial cells (HBMEC)

Primary isolates of HBMEC were cultured as previously described [70]. HBMEC were routinely grown in RPMI 1640 supplemented with 10% heat-inactivated fetal bovine serum, 10% Nu-serum, 2 mM glutamine, 1 mM sodium pyruvate, penicillin (100 units/ml), streptomycin (100 µg/ml), essential amino acids, and vitamins. The cells were incubated at 37°C in a humidified incubator with 5% CO_2_. Before each experiment, the culture medium was replaced with experimental medium containing Hams-F12/M199 (1∶1, v/v), supplemented with 5% heat-inactivated fetal bovine serum.

### *C. neoformans* association and transmigration assay

Total yeast cells associated with HBMEC were determined as previously described [41], [71]. Briefly, HBMEC were grown in 24-well tissue culture plates (or Transwell tissue-culture inserts with a pore diameter of 8.0 µm (Corning Costar)) until confluence. Inocula of 10^5^ *Cryptococcus* cells in experimental medium were added to each well (or top compartments in Transwells), and then incubated for 3 hr at 37°C. Free unbound yeast cells were removed by washing 3 times with PBS. The HBMEC were lysed with sterile distilled water and the lysates were diluted and plated onto sheep blood agar plates. The colonies were counted and results were presented as the total number of yeast cells per monolayer. Each set was triplicated and repeated at least three times independently.

The *in vitro* human blood-brain barrier (BBB) model was generated and used for fungal transmigration assays as previously described [70]. HBMEC were seeded on Transwell polycarbonate tissue-culture inserts with a pore diameter of 8.0 µm (Corning Costar) and cultured until their transendothelial electrical resistance (TEER) reached over 350 Ω/cm^2^, as measured by an Endohm volt/ohm meter in conjunction with an Endohm chamber (World Precision Instruments). The medium was replaced with experiment medium before each experiment. Yeast cells were washed with phosphate-buffered saline (PBS) and resuspended in HBMEC culture medium. 10^5^ *Cryptococcus* cells were added to the top compartment and then incubated at 37°C. At 3, 6, and 9 hr, the medium in bottom compartments was collected and immediately replaced with fresh medium. Fungal cell numbers in the collected medium were addressed by CFU counts to determine the number of transmigrated viable yeast cells. To determine the specificity of myo-inositol, the transmigration assay was performed in the presence of myo-inositol, scyllo-inositol, D(+) galactose or D(+) mannose (1 mM each) in the bottom compartments prior to addition of *Cryptococcus* cells (10^5^) in the top compartment of Transwells. Results are presented as the total number in the bottom chamber. Each set was triplicated and repeated three times independently. The statistical analysis of the data from our *in vitro* studies was done with a two-tailed Student *t* test. Statistical significance was determined at *P*<0.001.

### Measurement of myo-inositol concentration

The inositol concentration in medium was determined as previously described with minor modifications [46]. Briefly, the medium containing inositol collected from the top compartments of the BBB model was incubated with hexokinase to reduce interference from glucose by phosphorylation. The mixture was then incubated with 4.1 U/ml myo-inositol dehydrogenase for 15 min. Subsequently, 100 µl medium was mixed with an equal volume of detection reagent. The inositol concentration was determined by measuring optical density at 492 nm with a microplate reader (BioTek, Winooski, VT). Each assay was triplicated and repeated three times independently.

### *In vivo* virulence studies

Virulence of the *C. neoformans* strains was assessed using both a murine intravenous infection model and a rabbit CSF model of cryptococcosis as previously described [22], [72], [73]. For virulence study in a murine intravenous injection model, *Cryptococcus* strains were grown at 30°C overnight and cultures were washed twice with 1× PBS buffer by centrifugation, and resuspended at a final concentration of 5×10^5^ cells/ml. Groups of 15 female A/JCr mice (NCI-Frederick, MD) were used for each infection. Mice were infected with 5×10^4^ yeast cells of each strain in 100 µl PBS through tail vein injection [22], [74]. At each time point, 3 mice infected with either H99 or the mutant were sacrificed after 1, 6, 24, 48, and 72 hr post-infection. Fungal burden in infected brains was analyzed by CFU counts. Data from the murine experiments were statistically analyzed between paired groups using the long-rank test and the PRISM program 4.0 (GraphPad Software) (P values of <0.01 were considered significant). For the murine intracerebral injection model, mice were sedated with a Ketamine-Xylazine combination and the top of the head was sterilized using antiseptic. A total of 500 yeast cells in 50 µl were directly injected into the cerebrum as previously described [22]. For Rabbit infection, male New Zealand White (NZW) rabbits were treated with cortisone via daily injection and intrathecal inoculation into the subarachnoid space with 10^8^ cells of each *C. neoformans* strain (3 rabbits per group). Fungal burden in brain was analyzed by CFU counts.

### Microarray analyses for genes regulated by inositol

H99 overnight culture was washed with dH_2_O twice. Equal amount cells were inoculated on SD medium with or without 5 mM inositol and incubated for 24 hr before cells were collected for total RNA purification. Total RNAs were extracted using Trizol Reagents (Invitrogen) and purified using with Nucleospin RNA cleanup kit (Clontech, Mountain View, CA). Cy3 and Cy5-labeled cDNA were generated by incorporating amino-allyl-dUTP during reverse transcription of 5 µg of total RNA as described previously [75] and competitively hybridized to a JEC21 whole-genome array generated previously at Washington University in Saint Louis. After hybridization, arrays were scanned with a GenePix 4000B scanner (Axon Instruments) and analyzed by using GenePix Pro version 4.0 and BRB array tools (the National Cancer Institute, http://linus.nci.nih.gov/BRB-ArrayTools.html) as described previously [76]. The original microarray data was provided as supplementary file (Table S1) and also was submitted to GEO database (GSE41211).

To confirm the microarray results, we measured the mRNA levels of 6 genes under different conditions via quantitative real-time PCR (qPCR). First strand cDNAs of the purified RNAs were synthesized using a Superscript III cDNA synthesis kit (Invitrogen, Grand Island, NY) following the instructions provided by the manufacturer. Expression of candidate genes and *GAPDH* were analyzed with the comparative C_T_ method using SYBR green QPCR reagents (Clontech) as described previously [23].

### Detection of phospholipids in *Cryptococcus* using Thin Layer Chromatography (TLC) analysis

Overnight cultures of *Cryptococcus* were washed with dH_2_O twice and re-inoculated to 5 ml SD with or without 5 mM inositol and shaken for 2 hrs; 25 µCi/ml of ^32^P-phosphorus was added and the cultures were incubated with shaking overnight. Yeast cells were collected and concentrations were determined by hemocytometer counts. Steady-state labeling of phospholipid with ^32^P_i_ and lipid extraction were performed as described previously [77]. Lipids were dried in a SpeedVac apparatus, and resuspended into 500 µl chloroform. Five microliter aliquots were removed to measure the radioactivity in a scintillation counter. The remaining lipids were dried and frozen at −80°C. The individual phospholipids were resolved using two-dimensional silica gel TLC plates (EMD, Rockland, MS) using chloroform/methanol/ammonium hydroxide/water (90∶50∶4∶6, v/v) as the solvent system for dimension one and chloroform/methanol/glacial acetic acid/water (32∶4∶5∶1, v/v) as the solvent system for dimension two. The identity of the labeled lipids on thin-layer chromatography plates was confirmed by comparison with standards after exposure to iodine vapor. Radiolabeled lipids were visualized by phosphorimaging analysis with a Storm PhosphorImager (GE, Pittsburgh, PA). The relative quantities of labeled lipids were analyzed using ImageQuant software.

### Assay for hyaluronic acid production

The hyaluronic acid enzyme-linked immunosorbent assay (ELISA) kit (Corgenix, Denver, CO) was used to assay hyaluronic acid. The ELISA for hyaluronic acid production was followed the method as previously described with a few modifications [53]. Yeast cells (10^7^ cells) in the exponential-growth phase were incubated in individual wells at room temperature to trap the surface polysaccharide. After 60 min, the wells were washed with washing buffer carefully according to the manufacturer's instructions. A second solution containing a hyaluronic acid-binding protein-HRP conjugate was added to the wells and incubated for 30 min before adding substrates. The intensity of the resulting color was measured in optical density units with a spectrophotometer at 450 nm. The concentrations of hyaluronic acid were calculated by comparing the absorbance of the sample against a reference curve prepared from the reagent blank and hyaluronic acid reference solutions. The statistical significance was assessed by a 2-pair student t-test.

### Immunofluorescence microscopy

HBMECs were grown on coverslips coated with type-I collagen from rat tail (Millipore, Billerica, MA) until confluence. After incubation with *C. neoformans* (H99) for 1 hr, HBMECs were washed three times with PBS and processed for immunofluorescent staining as previously described [78]. Briefly, the cells were fixed with 4% paraformaldehyde for 30 min, permeabilized with 0.5% Triton X-100 for 5 min and then incubated with ZO-1 antibody, followed by AlexaFluor 488-conjugated secondary antibody (Invitrogen) to visualize. The coverslips were mounted with Vectashield mounting solution with DAPI (Vector Laboratory, Burlingame, CA) and observed with a Nikon fluorescence microscope. Images were taken with a MetaMorph Microscopy Automation & Image Analysis Software.

Analysis of fungal infection in mouse brain using confocal fluorescent microscope was performed as reported previously with modifications [79]. Thirty-fifty microns paraffin brain tissue sections were de-paraffined, subjected to antigen retrieval and permeabilized with 0.01% Triton X-100. Tissue sections were washed three times in PBS and incubated in blocking solution (5 mM EDTA, 1% fish gelatin, 1% essentially Ig-free BSA, 2% human serum and 2% horse serum) for 60 min at room temperature. Tissue sections were incubated in the proper diluted primary antibody (anti-GFAP, 1∶500, or anti-GXM, 1∶1000, provided by Dr. Arturo Casadevall, Albert Einstein College of Medicine) overnight at 4°C. Samples were washed several times with PBS at room temperature and incubated with the appropriate secondary antibodies conjugated to FITC or Cy3 for 2 hr at room temperature, followed by another wash in PBS for 1 hr. Tissue sections were then mounted on slides and stained with DAPI, and the cells were examined by a SP2 confocal microscopy (Leica). To identify and observe the tissue lesions induced by *Cryptococcus*, optical sections were acquired and reconstituted to focus in the lesion in these thick tissue sections. Images were analyzed with the NIS Elements Advance Research Program (Nikon). Antibody specificity was confirmed by replacing the primary antibody with a non-specific myeloma protein of the same isotype or non-immune serum.

## Supporting Information

Figure S1 **The** ***Cryptococcus*** **-HBMEC interaction does not change the integrity of the monolayer based on the transendothelial electrical resistance.** The *in vitro* human BBB model was prepared and incubated with *Cryptococcus* (H99 or the *itr1aΔ itr3cΔ* double mutant) in the absence (−) or presence (+) of inositol for 9 hr. Transendothelial electrical resistance (TEER) was measured with endohm/EVOM as described in transmigration assay. Each set was triplicated.
(TIF)

Figure S2 **The** ***itr1aΔ itr3cΔ*** **double mutant exhibits normal phagocytosis and intracellular growth inside macrophages.** Phagocytosis assays were performed in 48-well plates containing 5×10^4^ J774 macrophages. Total 2×10^5^ PBS washed *C. neoformans* strains H99, an *itr1aΔ itr3cΔ* double mutant and its complemented strain were added to the macrophages and incubated for 2 hrs at 37°C in 10% CO_2_. Extracellular yeast cells were removed by washing the wells with fresh DME medium. Macrophages containing *Cryptococcus* were further incubated for 0, 2, and 22 hrs before macrophages were lysed by dH_2_O. Yeast cell suspensions were plated on YPD for CFU counts to determine the total alive yeast cells. Error bars indicate the standard deviations.
(TIF)

Figure S3 **Verification of** ***Cryptococcus*** **genes upregulated by inositol treatment in a microarray analysis.** (A) Classification of genes upregulated by inositol treatment. Number indicates the number of genes in each class. (B) qRT-PCR was performed to confirm the upregulation of selected genes by inositol treatment identified in the microarray. Expression of candidate genes and *GAPDH* were analyzed with the comparative C_T_ method using SYBR green QPCR reagents (Clontech).
(TIF)

Figure S4 ***Cryptococcus*** **growth in the presence of inositol transporter inhibitor dinitrophenol.** *C. neoformans* (H99) were incubated with either DMSO, dinitrophenol (DNP) (0.3 mM) or phloretin (0.3 mM). After 30 min incubation, H99 cells were washed with experiment medium to remove inhibitors. Subsequently, washed H99 were added to experiment medium and the growth curves were generated up to 6 hr by measuring optical density at 600 nm. Each assay was set up in triplicate and independently performed three times.
(TIF)

Table S1 **Microarray analysis of genes regulated by inositol.** Wild type strain H99 was cultured in SD medium with or without 5 mM inositol for 24 hrs before RNAs were purified for *Cryptococcus* whole genome microarray analysis.
(XLSX)
