# Supplementary figures and images for: Brain Inositol Is a Novel Stimulator for Promoting Cryptococcus Penetration of the Blood-Brain Barrier
Source: PLoS Pathog. 2013 Apr 4;9(4):e1003247. doi: 10.1371/journal.ppat.1003247 (PMC3617100; doi:10.1371/journal.ppat.1003247)

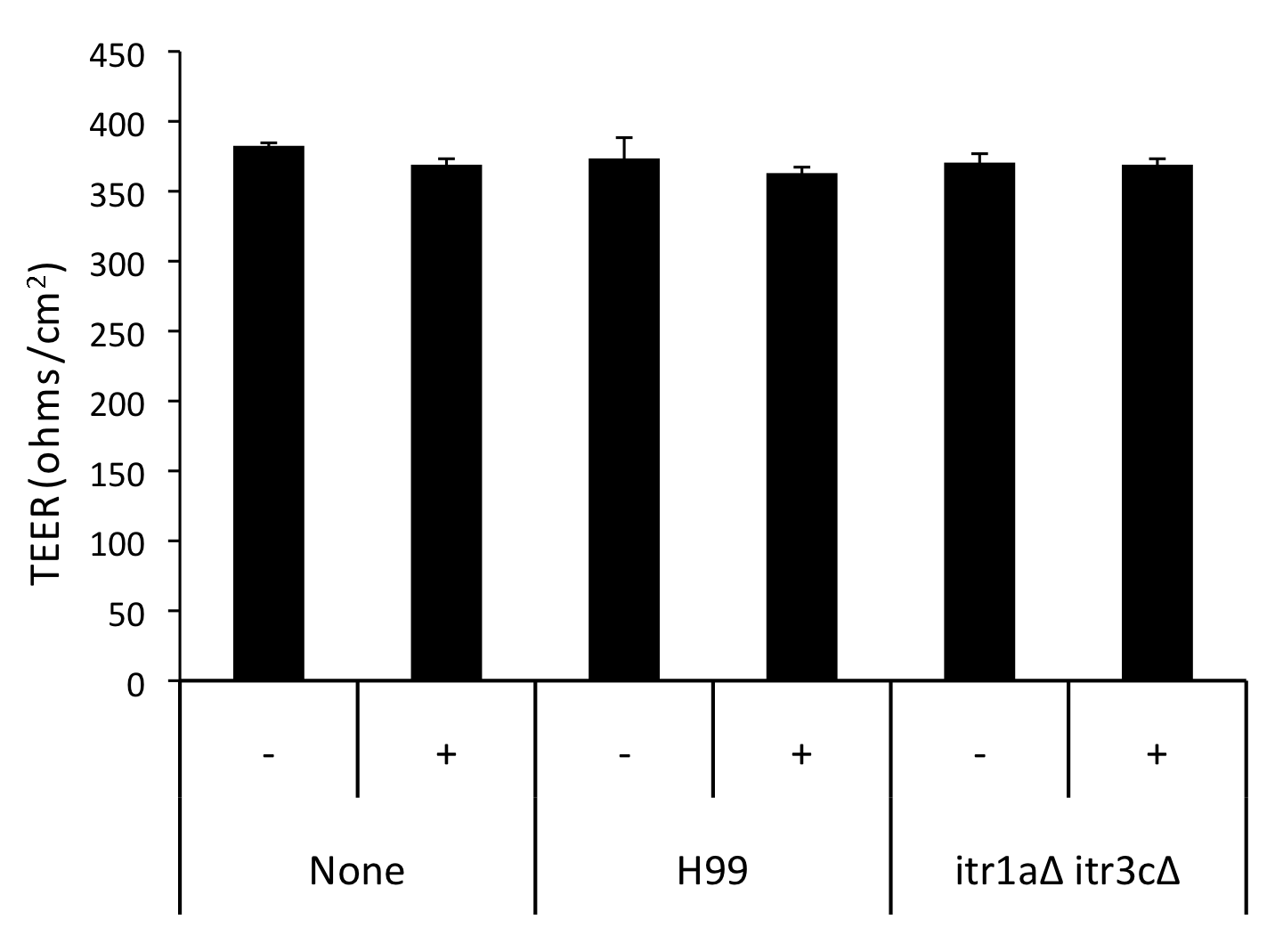

Supplement: Figure S1 — The Cryptococcus -HBMEC interaction does not change the integrity of the monolayer based on the transendothelial electrical resistance. The in vitro human BBB model was prepared and incubated with Cryptococcus (H99 or the itr1aΔ itr3cΔ double mutant) in the absence (−) or presence (+) of inositol for 9 hr. Transendothelial electrical resistance (TEER) was measured with endohm/EVOM as described in transmigration assay. Each set was triplicated. (TIF) [file ppat.1003247.s001.tif]

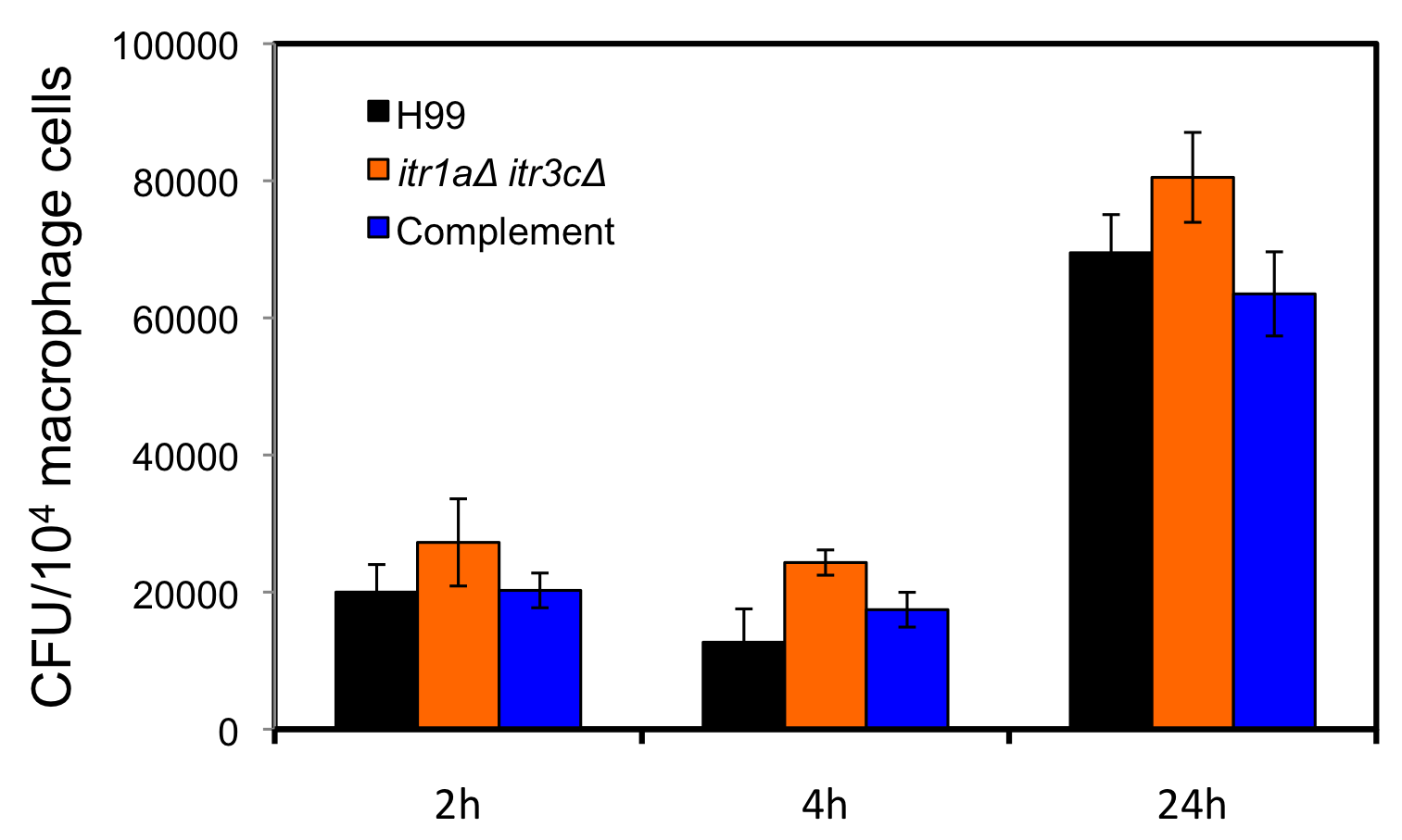

Supplement: Figure S2 — The itr1aΔ itr3cΔ double mutant exhibits normal phagocytosis and intracellular growth inside macrophages. Phagocytosis assays were performed in 48-well plates containing 5×10^4^ J774 macrophages. Total 2×10^5^ PBS washed C. neoformans strains H99, an itr1aΔ itr3cΔ double mutant and its complemented strain were added to the macrophages and incubated for 2 hrs at 37°C in 10% CO2. Extracellular yeast cells were removed by washing the wells with fresh DME medium. Macrophages containing Cryptococcus were further incubated for 0, 2, and 22 hrs before macrophages were lysed by dH2O. Yeast cell suspensions were plated on YPD for CFU counts to determine the total alive yeast cells. Error bars indicate the standard deviations. (TIF) [file ppat.1003247.s002.tif]

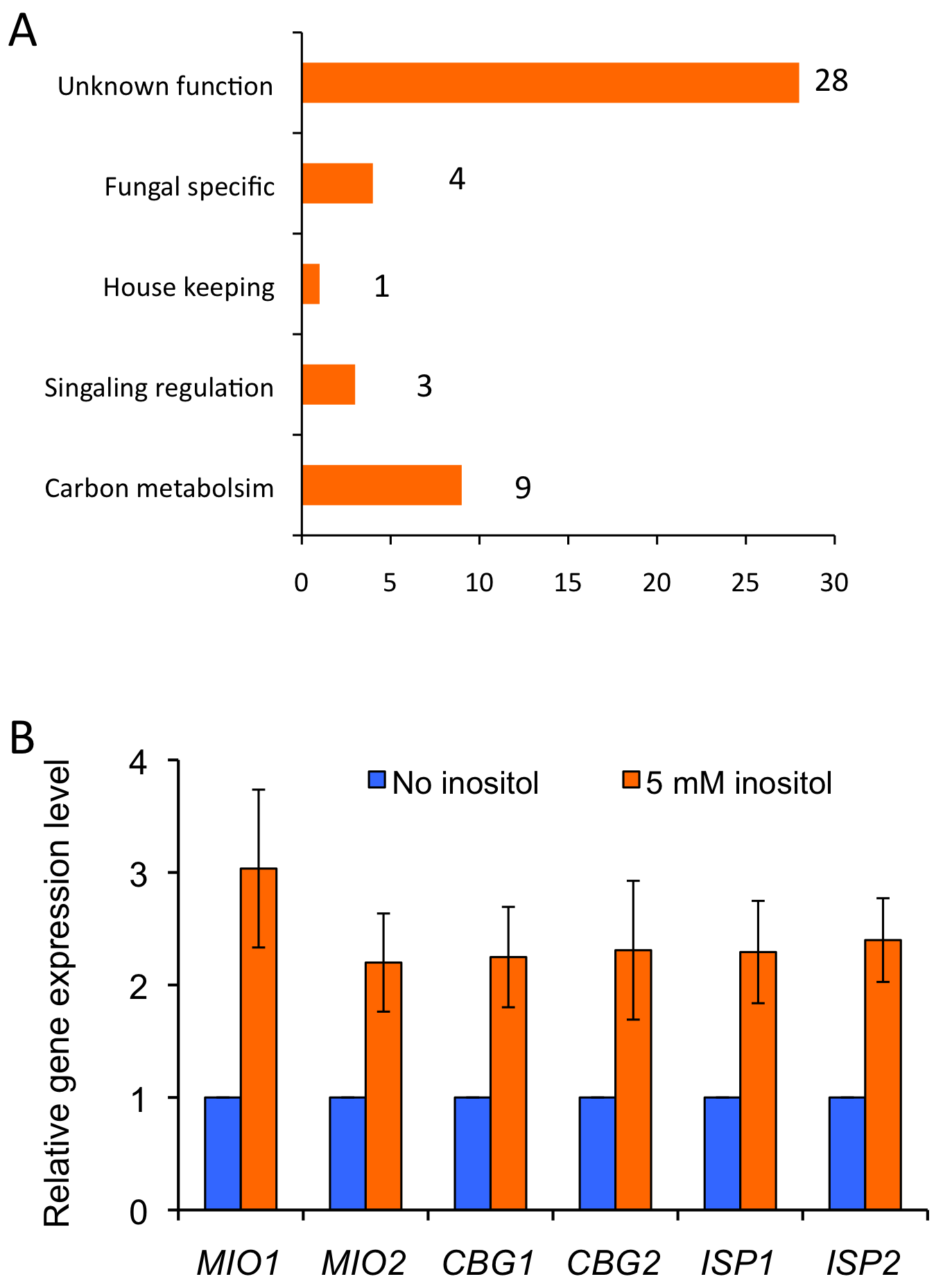

Supplement: Figure S3 — Verification of Cryptococcus genes upregulated by inositol treatment in a microarray analysis. (A) Classification of genes upregulated by inositol treatment. Number indicates the number of genes in each class. (B) qRT-PCR was performed to confirm the upregulation of selected genes by inositol treatment identified in the microarray. Expression of candidate genes and GAPDH were analyzed with the comparative CT method using SYBR green QPCR reagents (Clontech). (TIF) [file ppat.1003247.s003.tif]

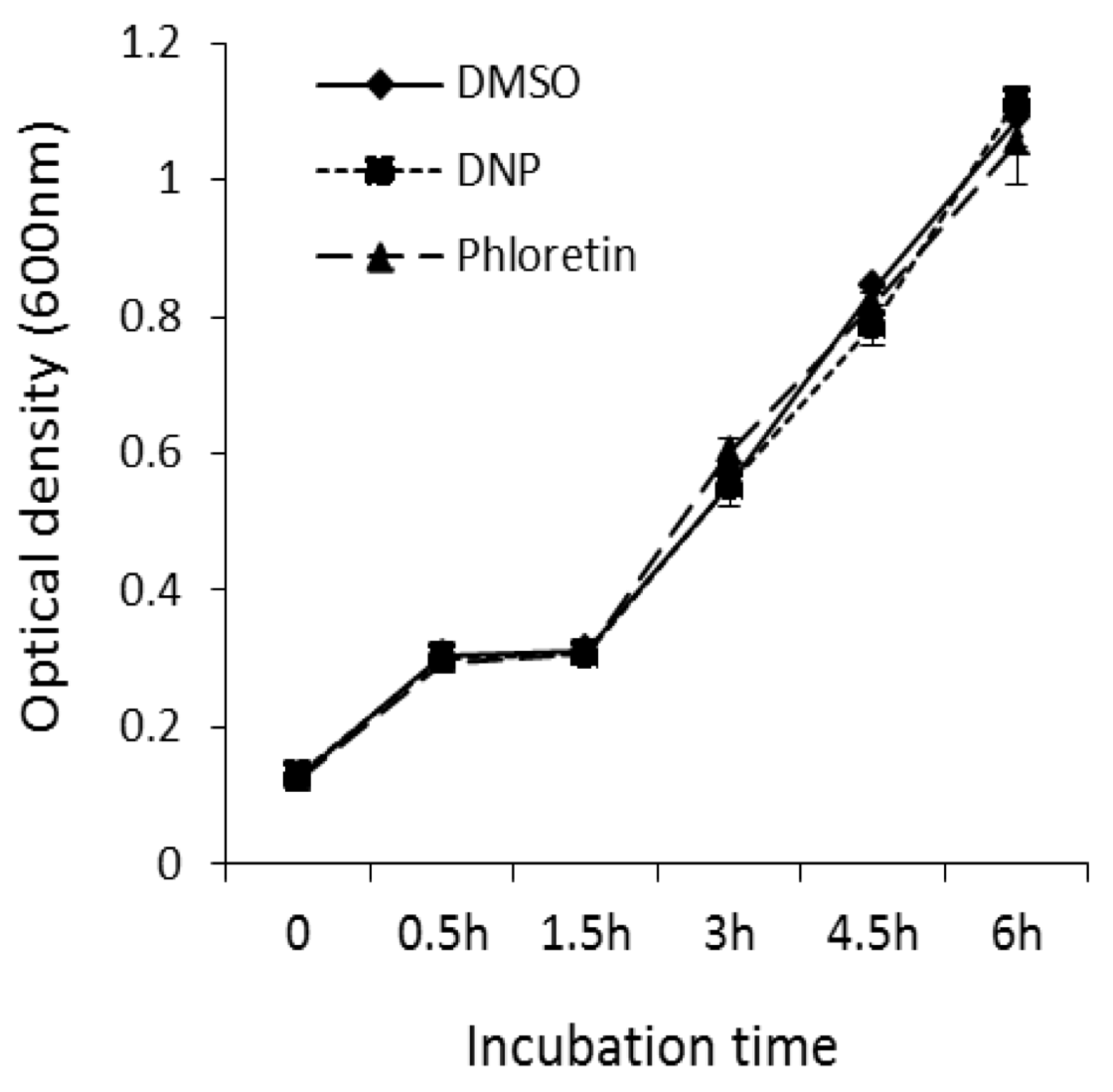

Supplement: Figure S4 — Cryptococcus growth in the presence of inositol transporter inhibitor dinitrophenol. C. neoformans (H99) were incubated with either DMSO, dinitrophenol (DNP) (0.3 mM) or phloretin (0.3 mM). After 30 min incubation, H99 cells were washed with experiment medium to remove inhibitors. Subsequently, washed H99 were added to experiment medium and the growth curves were generated up to 6 hr by measuring optical density at 600 nm. Each assay was set up in triplicate and independently performed three times. (TIF) [file ppat.1003247.s004.tif]
